# A New Method and Set of Parameters for Evaluating the Cushioning Effect of Shoe Heels, Revealing the Inadvertent Design of Running Shoes

**DOI:** 10.3390/bioengineering12050467

**Published:** 2025-04-28

**Authors:** Franz Konstantin Fuss, Tizian Scharl, Niko Nagengast

**Affiliations:** 1 Chair of Biomechanics, Faculty of Engineering Science, University of Bayreuth, D-95440 Bayreuth, Germany; tizian.scharl@ipa.fraunhofer.de (T.S.); niko.nagengast@uni-bayreuth.de (N.N.); 2 Department of Biomechatronic Systems, Fraunhofer Institute for Manufacturing Engineering and Automation IPA, D-95447 Bayreuth, Germany

**Keywords:** shoe testing, energy absorption, ratio of energy to force, shoulder point, classification of shoe design, development of test standard, ranking of cushioning properties, company trends, bioengineering of running shoes, biomechanics of running

## Abstract

According to standards, the heel soles of running shoes are currently tested with an energy absorption of 5 J. This study offers an alternative method to improve the measurement of cushioning properties. The new method uses the ratio of absorbed energy to applied force and determines the maximum of this ratio (optimum or shoulder point) and the associated optimal force, energy, and displacement. This method was applied to 112 shoe models using compression testing. The method was found to be insensitive to strain rates and identified shoes that were over-, well-, or under-designed (running before, at, or after the shoulder point, respectively) relative to the range of the first ground reaction force peak (0.700–2 kN). The optimum ratio was between 0.6 J/kN (barefoot shoes) and 11.2 J/kN (Puma RuleBreaker), the optimal energy was between 0.5 and 40.6 J, the optimal force was between 0.1 and 4.6 kN, and the optimal displacement was between 3 and 23 mm. Participants ran at or near the shoulder point (within the design forgiveness range) unless they were too heavy and ran at their preferred running speed. This study proposes replacing current standards with the new method, allowing consumers to make informed decisions regarding injury prevention while running.

## 1. Introduction

Daily physical activity should be an integral part of modern lifestyle, as it “*provides significant physical and mental health benefits*” [1] and contributes to the prevention of chronic diseases such as cardiovascular disease, cancer, and diabetes [1]. In 2022, almost one third (31%) of the world’s adult population was physically inactive [2]. The WHO recommends that “*adults should do at least 150–300 min of moderate-intensity aerobic physical activity; or at least 75–150 min of vigorous-intensity aerobic physical activity per week*” [3]. These activities include running. Running is not restricted to special sports facilities (unlike, e.g., climbing, swimming, and other gym activities) and does not require any special sports equipment other than ordinary running shoes. The popularity of running shoes is reflected in the global revenue of the three major athletic footwear giants (Nike, Adidas, and Puma), which totalled $47 billion in 2022 [4].

Running shoes must be designed to alleviate, if not prevent, acute injuries and chronic overuse/overstrain syndromes. In fact, the one-year injury incidence among recreational runners has been reported to be about 45% [5,6], with approximately 50% of all injuries occurring in the knee and calf muscles [6] or in the foot/ankle and knee [5]. In particular, the heel segment of running shoes must be able to absorb sufficient energy during heel strike that would otherwise be absorbed by the leg. This expectation may only apply to heel strikers and not to midfoot strikers; however, it has been reported [7] that observational data found that 94% of all investigated runners were heel strikers, while self-reported data showed that only 32% of all investigated runners considered themselves heel strikers.

The design and development of running shoes should therefore be an integral bioengineering process comparable to the development of orthoses and prostheses, based on biomechanical data from test persons, and material data from mechanical testing. The corresponding testing of running shoes for their properties and quality and the design of these tests are also bioengineering tasks, considering that their results will have an effect on the human body. These test data should be made available to customers to help them make informed decisions when selecting a running shoe, particularly for the prevention of running injuries. Subsequently, the current standard tests for athletic shoes are explained and reviewed.

ASTM (F1614-99 [8] and F1976-24 [9]) is the standard for testing the heel segment of athletic shoes. ASTM F1614-99 (1999; Standard Test Method for Shock Attenuating Properties of Materials Systems for Athletic Footwear) offered three procedures [8]:

- *A for falling weight impact machines*
- *B for compression force-controlled machines, and*
- *C for compression displacement-controlled machines.*

ASTM F1976-24 (2024; Standard Test Method for Impact Attenuation of Athletic Shoe Cushioning Systems and Materials [9]) was limited to procedure A.

The main feature common to both ASTM versions is that the standard or nominal value of the reference maximum total energy input applied by this test method is set at 5 J. The rationale for this method is explained in Note 3 of ASTM F1614-99 [8]: “*There is no evidence to support comparisons of data for tests which used … different reference maximum energy applied values…*”. However, the reason for the specific value of 5 J was not explained. Another method for testing shoes (DIN EN 12743:1999; Footwear—Test methods for outsoles—Compression energy [10]) uses a maximum force set at 5000 N and a deflection rate at 0.167 mm/s.

Other test standards for shock-absorbing materials use the same approach:

- ISO 3386-1:1986 (Polymeric materials, cellular flexible—Determination of stress–strain characteristics in compression [11]) serves to compare stress data of foams at a strain value set to 40% (“CV_40%_”).
- ASTM D3574 C (Foam Force Deflection Testing, ASTM, [12]) “*specifies a method for measuring the compression force necessary to produce a 50% deflection … of the foam test specimen. … This test is very similar to ISO 3386, but with slight technical differences that prohibit a direct comparison*” [13].

The criticism of directly comparing results obtained using different methods or even different standards is reflected in note 3 of ASTM F1614-99 [8]. This principle justifies the need to set reference test parameters with a fixed value. The counterargument for a different approach, however, is that runners do not apply exactly 5 J of energy to the heel segment during heel or foot strike, nor does the optimal point of shock absorption lie exactly at 40% or 50% of the strain. Energy absorption models such as the buckling element or the pneumatic element (airbag), for example, have their optimum at 50% or 68.38% of the strain, respectively [14]. The maximum optimal strain range determined by Fuss [14] for 88 different shock-absorbing materials and structures was 48.5–81%.

Nevertheless, there are several literature sources that used ASTM F1614-99 [8] or ASTM F1976-24 [9] to test athletic shoe heels. Delattre et al. [15] used ASTM F1614 and concluded that results are not comparable when using different variables and test methods. Delattre and Cariou. [16] used ASTM F1976 to mechanically characterise shoes. They discovered a relationship that allowed them to predict 75% of the variance in “heel penetration” (shoe cushioning in industry-defined terms) from women’s perception of running, using a mechanical parameter, namely the absolute maximum shoe compression value. Determan et al. [17] used ASTM F1614-99 and studied the impact energy generated by skateboarders, which can be as high as 40–50 J. Accordingly, they applied an alternative impact test procedure at 44 J. Their results showed that the shoe that responded best to 5 J impacts performed worst under 44 J impacts. Conversely, the shoe that performed worst under 5 J impacts responded best to 44 J impacts. The authors recommended caution when applying standard low-impact methods to athletic shoes. They criticised that “*testing methods and standards such as ASTM F1614 may be appropriate for low-load activities such as walking or running, but provide a poor indicator for shock attenuation capability for higher-impact sports such as skateboarding*”. Finally, they suggested that “a new test method should be developed for evaluating the shock attenuation properties of footwear for sports where athletes experience relatively high impact forces to their feet”, but without quantifying the actual impact energy experienced when running at different speeds.

Lloria-Varella et al. [18] used ASTM F1614-99 and detected significant changes in shoe properties such as reduced thickness and increased stiffness after running with the individual running shoes. For example, the heel sole thickness decreased from pre-race to post-race (38 km), while stiffness and peak force increased, and energy loss decreased.

Shorten [19] used the ASTM F1976-13 impact test with the prescribed total impact energy of 5 J in three marathon racing shoes. The author investigated peak compression and force, as well as energy loss among other energy parameters, and concluded that measurement results are sensitive to variations in the test protocol.

Schwanitz et al. [20] compared three methods, namely ASTM F1614 Procedure A, ASTM F1614 Procedure B (energy set at 5 J for both methods), and the HIT method (hydraulic impact test [21]) with a peak force set at 1500 N. The authors criticised that the results of the three methods were not comparable because the HIT method produced an impact energy of 8.11 J compared to preset energy of 5 J for ASTM F1614 [8,9]. Similarly, the peak forces of ASTM F1614 procedures A and B were 992 N and 985 N, respectively, compared to the preset peak force of 1500 N for HIT.

Pisciotta and Shorten [22] used ASTM F1976 to calculate a “*peak impact shock score*” (maximum deceleration in *g*; *g*_max_), which corresponds to peak impact forces of 809–1259 N (calculated from Pisciotta and Shorten’s data [22]), at 5 J of energy input. As a result of their user experience study, after approximately one month and 160 km of running, the shoe testers reported their overall rating of the shoe as well as their ratings of specific shoe characteristics, including perceptions of heel and forefoot cushioning, from “low” to “high” on a seven-point Likert-type rating scale. Pisciotta and Shorten’s results [22] showed that there was no significant difference between the ranks of four shoes with average ranks between 5 and 6, while the fifth shoe was rated significantly lower than the other four shoes, averaging 3.8. These ratings corresponded to *g*_max_ of 9.7–12.6 g for the first four shoes compared to an average *g*_max_ of 15.1 g for shoe number five. This result suggests that participants rate shoes lower when they are associated with a greater impact force.

Based on the results from literature sources using ASTM standards F1614-99 [8] and F1976-24 [9], as well as the critical statements of the authors, the following knowledge gaps can be defined:

- Gap 1: Current standards and methods for assessing shoe heel mechanics rely either on compression with a constant and standardised energy level (e.g., 5 J [8,9]) or with a constant force (e.g., 5000 N [10] or 1500 N [21]). The reason for this choice is the comparability of test results. However, it is a fact that runners do not run with the same energy level or the same force level. An equal energy level can be ruled out a priori, as heels of different thicknesses absorb different amounts of energy at the same force. The obvious knowledge gap in this context is the search for an alternative method. The contribution to the literature and lateral innovation of an alternative method could be that it is independent of energy and force levels and yet suitable for comparing test results using new reference parameters.
- Gap 2: It is well known in the literature that energy-absorbing materials have an optimal energy absorption point, namely the maximum ratio of absorbed energy to applied force. The knowledge gap in athletic footwear, particularly running shoes, is that this approach has not previously been used to evaluate and assess the mechanics of the heel segment of shoes. The contribution to the literature and the innovation of this study are the application of the optimal point method to shoe heel testing and the use of the test results to evaluate energy absorption relative to the load applied to the heel.

The aim of this study was therefore to develop a new concept and method to measure the cushioning potential of the heel of an athletic shoe independent of preset energy [8,9] or force values [10,21]. The force data or “peak impact shock score” data obtained from the current standards are only valid when runners run at 5 J compression energy at the heel.

The new concept and method developed in this study are applied to experimental aspects and conditions as well as to a variety of different athletic shoes to understand the potential and the limitations of the method for future shoe testing protocols.

## 2. Materials and Methods

### 2.1. Rationale of the Methods Used in This Study

While ASTM standards [8,9] take care of the proper impact scenario when loading the heel, the preset level of 5 J actually prevents the direct comparison of different shock-absorbing materials and structures. These shock absorbers are highly non-linear. Fixed and inflexible testing conditions are only appropriate for linear materials. Based on these issues, this study proposes to compare the damping parameters of shoe heels at the individual optimum points of energy absorption.

**Figure 1:**
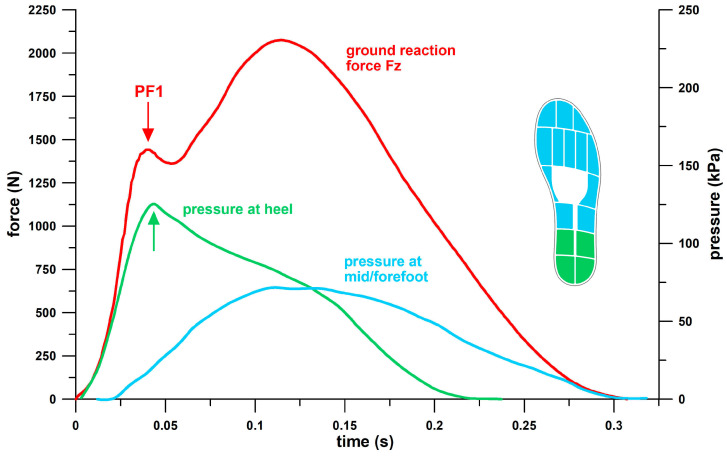
Vertical ground reaction force Fz (force plate data) and foot sole pressure (Moticon insole, Moticon ReGo AG, Munich, Germany) vs. time of a participant running with a heel strike pattern at 10 kph; the pressure data were averaged (weighted by sensor areas) across the heel and mid/forefoot areas; the arrows indicate the alignment of Fz and peak heel pressure data; PF1: peak force 1, i.e., first force peak of the Fz force.

This optimum point, also referred to as the “shoulder point” [14,23,24], is found at the peak ratio of energy per unit volume (*W*) to stress (*σ*) in materials, and energy-absorbing structures exhibit this peak ratio of energy (*E*) to force (*F*). If an energy-absorbing structure is loaded with less force than the optimum force *F*_opt_ at the shoulder point, the structure will feel too hard due to insufficient compression. If a structure is loaded with more force than the optimum force *F*_opt_ at the shoulder point, the compression level would suggest a softer feel, but the structure is already compressed in the densification regime, bottoming out, and therefore feels too hard. Between these two extreme conditions, i.e., the optimum force *F*_opt_ before or after the shoulder point, the structure is expected to provide a “better” feel. In design terms, the two extreme conditions can be described as over- and under-designed, respectively. This method, previously applied to energy-absorbing materials [14,23,24], namely mechanical elements considered as materials [14] and structures treated as materials [14,24], opens a new perspective when applied to the energy-absorption or cushioning potential of the heel sole of sports shoes, especially running shoes. Note that the terms “energy absorption” and “cushioning” used in this study refer to large peak ratios (maximum of *E*_opt_/*F*_opt_) at the shoulder point and to large energy absorption *E*_opt_ at the shoulder point, regardless of whether the users are actually running at the shoulder point. What the two terms, energy absorption and cushioning, do not refer to is the reduction of the peak force at the heel, previously incorrectly referred to as the shock spike [25], because the first peak of the vertical ground reaction force during running is not a transient spike but represents the full load experienced by the heel as a loadbearing structure (referred to in this study as the first peak force, *PF*1; Figure 1).

In contrast to this new method, the ASTM standard [8,9] misses much valuable information, as human runners generally do not run with heel–sole energy absorption of exactly 5 J. As shown in the Results section, different shoe models have different *E*_opt_/*F*_opt_ ratios, as well as different *F*_opt_ and *E*_opt_ values. The reason for this variety is often due to inadvertent designs, i.e., shoes not specifically designed for defined shoulder point parameters, as the shoulder point principle was not previously known in sports shoes.

The different aspects to be considered in this context were realised through various sub-studies focusing on specific topics and research questions:

- Are the shoulder point parameters affected by the compression speed? If they are insensitive to compression speed, can we use a single speed for testing?
- How are the shoulder point parameters related to each other? Can we identify shoes with optimal cushioning, design flaws, and shoe groups that are better suited for cushioning? Are the shoulder point parameters different for minimal and maximal shoes? Can we identify corporate design trends related to the shoulder point parameters?
- How many compression cycles do we need until a steady state of the shoulder point parameters is reached?
- What is the repeatability and uncertainty of shoulder point parameters?
- Does the shoe size affect the shoulder point parameters?
- Does the removable insole of the shoes affect the shoulder point parameters?
- Can we identify a design forgiveness parameter that provides approximately the same feeling when running with different body weight but the same shoe size?
- How is *PF*1 (formerly: shock spike) related to *F*_opt_, and can we identify under- and over-designed shoes?

Subsequently, the mechanics of shoulder point parameters are explained in detail.

### 2.2. Shoulder Point Parameters

If a function through the origin has a positive slope and the slope increases continuously, then the ratio of the integral of this function to the original function has a maximum. This is especially true for functions that asymptotically approach infinity, such as reciprocal, tangent, and circular functions, but also for x^x^, for example. When compressing materials or structures, the sample cannot be compressed more than its original thickness. This principle reflects the fact that the stress–strain curve asymptotically approaches infinity, actually even before the strain reaches unity. Functions such as exponentials or polynomials do not apply, since the ratio of the integral to the original function is either constant or a linear function, respectively.

Since the integral of the force *F* or stress *σ* with the deflection *x* or strain *ε* is the energy *E* or energy per unit volume *W*, the ratio of the integral to the original function is either *E*/*F* or *W*/*σ*, respectively. The maximum or peak value of these ratios is commonly referred to as the “shoulder point” ([14,23,24]). While the properties of materials are commonly reported based on the stress *σ*–strain *ε* curve, the non-normalised properties of shoes, particularly the heel segment of athletic shoes, are derived from the force *F*–deflection *x* curve. To avoid confusion, note that the first deflection or strain derivatives of *F* and *σ* are the stiffness *k* or the modulus *E*, respectively. As the energy *E* and the modulus *E* could be confused in this study, *E* primarily refers to energy unless explicitly referred to as modulus *E*.

**Figure 2:**
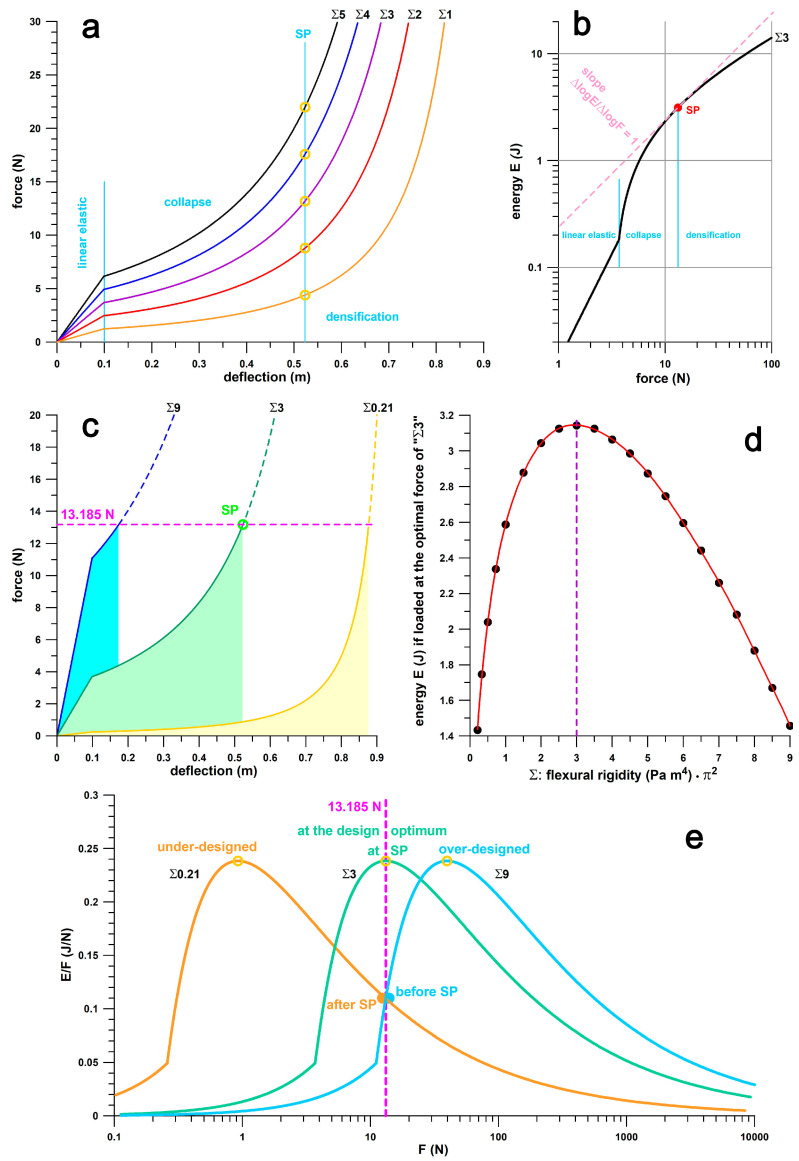
Shoulder point principle: (**a**) force vs. deflection, SP = shoulder point, Σ = flexural rigidity (Young’s modulus times the second moment of area) times π^2^ of the buckling element of the shock absorber; (**b**) energy vs. force, identification of the shoulder point from a tangent with slope =1; (**c**) force vs. deflection, the coloured areas under the curve correspond to the energy absorbed at a load of 13.2 N; Σ3 is loaded at the shoulder point; Σ9 is loaded before the shoulder point; Σ0.21 is loaded after the shoulder point; (**d**) energy E (absorbed up to a load of 13.2 N) vs. Σ; (**e**) ratio of energy E to force F versus force F; the three E/F vs. F curves are geometrically identical when plotted on a single logarithmic coordinate system (logF).

In this study, the key performance parameters of the heel of sports shoes are as follows:

(1) The maximum ratio of *E* to *F* is the ratio at the optimum point, also referred to as the shoulder point, which is used to define the remaining three parameters. Its symbol should be correctly written as (*E*/*F*)_max_, (= *E*_opt_/*F*_opt_; cf. below) but is shortened to *E*/*F*_max_ in this document. *E*/*F*_max_ is the optimum point of energy absorption, which is convincingly explained by Figure 2. The unit of *E*/*F*_max_ used in this study for shoes is joules per kilonewton (J/kN), which collapses to a displacement after cancellation of accelerations and masses. Note that this displacement is the ratio of *E* at the shoulder point to *F* at the shoulder point (i.e., Δ*E*/Δ*F*, where *E*_max_ of Δ*E* = *E*_opt_ and *F*_max_ of Δ*F* = *F*_opt_, and *E*_min_ of Δ*E* = 0 and *F*_min_ of Δ*F* = 0), and not the actual displacement *x* of the structure (where d*x* = d*E*/*F*, or *x* = ∫*F*^−1^ d*E*). The biomechanical significance of *E*/*F*_max_ in the context of athletic shoes lies in the principle that the energy absorbed by the human leg during barefoot running should be absorbed as much as possible by the heel of the shoe, with the least possible load on the heel. *E*/*F*_max_ provides the data for this principle.
(2) *F*_opt_, the force *F* of the structure at the shoulder point expressed in kilonewtons (kN). The biomechanical significance of *F*_opt_ in the context of athletic shoes lies in the fact that *F*_opt_ should be close to *PF*1 (Figure 1). The magnitude of *F*_opt_ opens the possibility of a design classification (under-, well-, over-designed heels).
(3) *x*_opt_, the displacement *x* of the structure at the shoulder point in millimeters (mm). The biomechanical significance of *x*_opt_ in the context of athletic shoes lies in the fact that the larger *x*_opt_ the more energy is absorbed (since the energy absorption of a Hookean spring is *E* = *kx*^2^/2, where *k* is the stiffness of the heel). *x*_opt_ is constrained by the design thickness of the heel. Minimalist shoes have a smaller *x*_opt_ than maximal shoes.
(4) *E*_opt_, the energy *E* absorbed by the structure at the shoulder point expressed in joules (J). The biomechanical significance of *E*_opt_ in the context of athletic footwear lies in the fact that, theoretically, there is no upper limit. The higher the *E*_opt_, the better. However, *E*_opt_ is limited by the thickness of the heel and the proximity of *F*_opt_ to *PF*1.

To illustrate the concept of the above key performance parameters, Figure 2 shows the shoulder point parameters of a theoretical energy absorber modelled as a buckling element [14] in series with a Hookean spring. The latter maxes out at *x* = 0.1 m, and the buckling element starts to buckle at the force generated at *x* = 0.1. Figure 2a shows the force–deflection curves of the energy absorbers whose range of flexural rigidity (*EI*, modulus *E* times second moment of area *I*) times π^2^ is 1 ≤ *Σ* ≤ 5 Pa m^4^ (where *Σ* = *EI* π^2^). Figure 2a shows the linear elastic phase enabled by the Hookean spring, the collapse phase after the onset of buckling, the shoulder point, and the densification phase after the shoulder point. The shoulder point is detailed in Figure 2b by plotting *E* vs. *F* on a double-logarithmic graph. The shoulder point, which is usually defined from *E*/*F*_max_, is identified here by the unique feature of d(log *E*)/d(log *F*) = unity [14]. If the energy absorber is loaded exactly at the shoulder point, *F*_opt_ equals 13.185 N in the example shown in Figure 2c. If the other energy absorbers with 0.21 ≤ *Σ* ≤ 9 are loaded with the same force (13.185 N) and evidently not at the shoulder point, but rather before or after, the energy *E* absorbed by the energy absorber (i.e., the area under the force–deflection curve) is smaller than the corresponding energy of the absorber with *Σ* = 3 (Figure 2c). The energy absorbed by all absorbers (0.21 ≤ *Σ* ≤ 9) versus *Σ* (Figure 2d) shows that the maximum energy is absorbed precisely at the shoulder point, compared to the energy absorbed before or after the shoulder point. The three absorbers of Figure 2c are shown on an *E*/*F*_max_ vs. *F*_opt_ plot in Figure 2e. The absorber with *Σ* = 3 is loaded at the shoulder point, while the absorbers with *Σ* = 0.21 and 9 are loaded after and before the shoulder point, respectively. Figure 2c, in combination with Figure 2d,e, illustrates the importance of the shoulder point, and the optimum energy absorption at the shoulder point. Absorber *Σ*9 is too stiff, with a steep linear-elastic phase, so it cannot absorb enough energy. The peak load of 13.185 N results in a deflection of only 174 mm and the absorber should feel hard well before the shoulder point. Absorber *Σ*0.21 is too soft, so it bottoms out quickly and reaches the densification phase where the force increases steeply. Despite a deflection of 873 mm, the absorber cannot absorb enough energy either, and would therefore feel just as stiff when bottoming out well after the shoulder point. Only the *Σ*3 absorber can store a decent amount of energy over a deflection of 523 mm, and is assumed to feel right, or soft compared to the other two absorbers. If the chosen absorber is to cushion a force of 13.185 N, the *Σ*0.21 absorber is comparatively ***under-designed*** (designed for smaller forces), the *Σ*9 absorber is ***over-designed*** (designed for larger forces), while the *Σ*3 absorber is at the ***design optimum***. If the designer is not familiar with the principles of optimum energy absorption and the shoulder point principle, the design is obviously an ***unwitting*** or ***inadvertent*** design rather than a strategic design.

**Figure 3:**
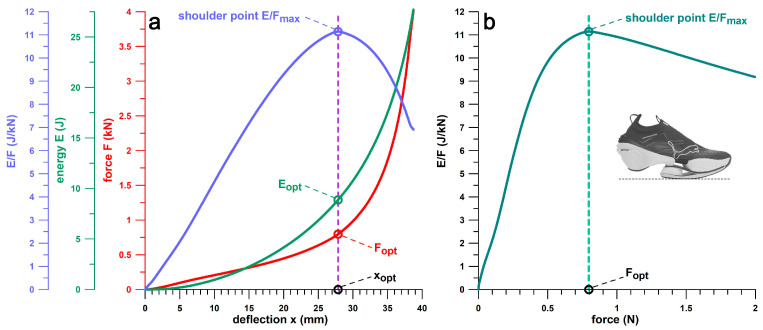
Shoulder point parameters exemplified by means of the PUMA RuleBreaker heel segment: (**a**) ratio of energy E to force F, energy E, and force F vs. deflection x; (**b**) ratio of energy E to force F versus force F; E/F_max_ = maximum ratio E/F at the shoulder point; E_opt_, F_opt_, x_opt_ = E, F, x at the shoulder point.

Figure 3 shows the derivation of the shoulder point parameters using the PUMA RuleBreaker shoe as an example. Figure 3a combines all 4 shoulder parameters in a single diagram. Figure 3b is particularly useful for the assessment of the design outcome, as it shows *E*/*F* versus force *F*. In the specific case of the PUMA RuleBreaker shoe, the optimum force at the shoulder point, *F*_opt_, is approximately 800 N. Although this force could correspond to a runner’s body weight, it is still in the *PF*1 range (cf. Section 2.12 and Section 3.8). However, the RuleBreaker shoe was developed for mid-foot strikers, and the heel segment does not touch the ground when standing. Even when running with a heel strike, it is very likely that the forces are distributed more between heel and midfoot than in other running shoes. In this case, *F*_opt_ is appropriate.

### 2.3. Test for Compression Speed Dependence of Shoulder Point Parameters

Different test methods, such as impact tests (e.g., ASTM F1614-99 [8], procedure A) and static testing (e.g., ASTM F1614-99 [8], procedure B) are expected to generate different results, primarily because the modulus *E* of a viscoelastic solid increases as a function of the strain rate [26], as does the stress *σ* and the energy per unit volume, *W*, at a given strain *ε*. However, at the optimum (maximum) ratio of *W* to *σ* (shoulder point), the modulus *E* as a multiplier of the stress–strain curve (*σ* = *E ε*) cancels out in the ratio *W*/*σ*.

Two samples (70 × 70 × 10 mm) of each of four different closed cell foams were tested: A: Pebax, 160 kg/m^3^; B: EVA Profoam, 210 kg/m^3^; C: Develite, 135 kg/m^3^; D: Pebax, 127 kg/m^3^. All four foams are suitable for the manufacture of soles for sports shoes. The test methods included a static compression test (10 cycles, triangular displacement profile, constant compression speed of 10 mm/s, maximum compression of 9 mm; Z050, ZwickRoell GmbH & Co. KG, Ulm, Germany) and a dynamic one (NMB/Schenck PL10N, New Materials Bayreuth, Bayreuth, Germany). The dynamic displacement profile was sinusoidal (10 cycles), but only half of the cycle amplitude was used for compression. The four foams were tested at different cycle frequencies *f* (0.83, 1, 1.67, 2.27, 2.78 Hz) and corresponding compression values *x* (A: 6–7.79 mm, B: 5–6.9 mm, C + D: 5.9–7.7 mm), resulting in compression speeds of 2π*fx* mm/s (A: 31.3–136.1 mm/s, B: 26.1–120.5 mm/s, C + D: 30.8–134.5 mm/s). The range of compression velocities (static, lowest velocities; and dynamic, five velocities) was therefore larger than one order of magnitude, which is sufficient to detect any velocity dependence [26]. The cycle frequencies and compression amplitude were chosen so that the compression period and peak force resemble those during running at different speeds.

The force–displacement data were converted to stress–strain data, and the following four parameters were extracted, based on the method explained above: maximum *W*/*σ* (optimum, shoulder point value), *ε* at optimum, *σ* at optimum, and *W* at optimum. The slowest dynamic speed had to be excluded because the corresponding displacement did not reach the shoulder point. The velocity dependence was tested with the Friedman rank sum test for correlated samples from five groups (static speed and four dynamic speeds), with the hypothesis that the four shoulder point parameters are not velocity dependent (*p*-value > 0.05). In the case of *p* < 0.05, the post–hoc Dunn–Bonferroni test was used to identify the significantly different groups.

### 2.4. Shoes Tested in This Study

We tested 112 shoe models (Table 1) from 6 main brands (Puma, Adidas, Asics, Brooks, Mizuno, and Nike) and 18 other brands in this study. Most of these were running shoes; some shoes were from other sports (basketball, handball, futsal); and two shoes were for hiking and walking.

**Cohort 1** consisted of shoes from Mizuno Oceania Corporation as part of the Mizuno-funded research grant “Kansei Engineering of Running Shoes” (RMIT University, 2010–2012). The specific testing method using shoulder point principles was not part of the funded project.

**Cohort 2** consisted of personal sports shoes of master’s students, tested during the laboratory unit of the “sports shoe technologies” courses within the “MSc in International Sports Technology” (RMIT University, Melbourne, Australia; 2012–2016) and “MSc in Sports Technology” (University of Bayreuth, Bayreuth, Germany; 2023–2025) programs.

**Cohort 3** consisted of 6 running shoes varying in their minimalist index studied for a PUMA funded project [27]: Vivobarefoot, Joe Nimble, Nike Free, Puma Liberate, Puma Velocity, and Puma Deviate Elite.

**Cohort 4** included shoes used for smaller research projects such as PUMA shoes (running, handball, and basketball), Brooks shoes, and prototype CEP Omnispeed shoes. The specific testing method described below was not part of these funded projects.

**Cohort 5** included shoes originally purchased for specific research projects and teaching purposes (Adidas MegaBounce, Adidas BounceTitan, Adidas SpringBlade), or received from PUMA (PUMA Rulebreaker, PUMA FastR), and were not related to a funded project.

**Figure 4:**
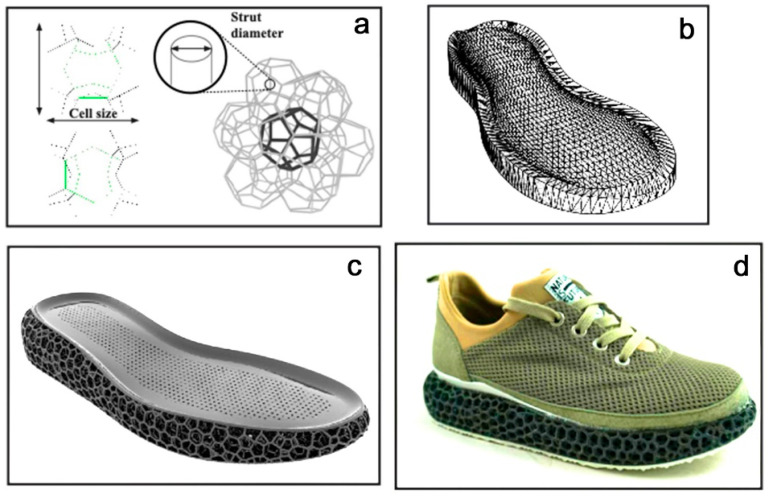
Development process of the shoes with 3D-printed midsoles: (**a**) Weaire–Phelan unit cells; (**b**) 3D model of the midsole; (**c**) 3D-printed midsole; (**d**) assembled experimental running shoe.

**Cohort 6** consisted of experimental shoes with additively manufactured shoe soles (Figure 4) with three different 3D-printing methods (LS, MJF, HSS), funded within the MONOMAT project (German Federal Ministry for Economic Affairs and Climate Action, Berlin, Germany). The specific test method described below was not part of the funded project, as per the funding application.

The integral 3D structure was an open-cell structure consisting of cell edges of a Weaire–Phelan foam. The computational tessellation of a Weaire–Phelan unit cell with a corresponding cell size and strut diameter was flexibly implemented by the CAD-scripting plug-in Grasshopper (McNeel, Seattle, WA, USA) into the mid-sole geometry. Two versions (1.1 and 1.2) were manufactured with cell sizes of 15 mm × 15 mm × 15 mm and 20 mm × 20 mm × 20 mm as well as strut diameters of 16 mm and 25 mm, respectively. Three different printing processes were used: Laser Sintering (LS), High-Speed Sintering (HSS), and Multi-Jet Fusion (MJF). Due to an extremely soft feel in the preliminary test, HSS 1.1 was eliminated from the test series in the following test scenarios. The laser-sintered (LS) and high-speed-sintered (HSS) midsoles were fabricated from Rolaserit TPU PB01 powder (AM Polymers, Willich, Germany) together with a 3D Systems Vanguard HS (Vanguard, Philadelphia, PA, USA) or VX 200 (Voxeljet, Friedberg, Germany), respectively. The MJF sole was manufactured from Ultrasint TPU1 powder (BASF, Ludwigshafen, Germany) via the MJF machine HP 5200 (HP, Palo Alto, Santa Clara, CA, USA). A conventional carbon rubber sheet, commonly used in running shoes, was glued to the underside of the 3D-printed midsole. A textile mesh based on polyester fibers was used for the shoe’s upper, which was glued to the midsole.

### 2.5. Test Method

**Figure 5:**
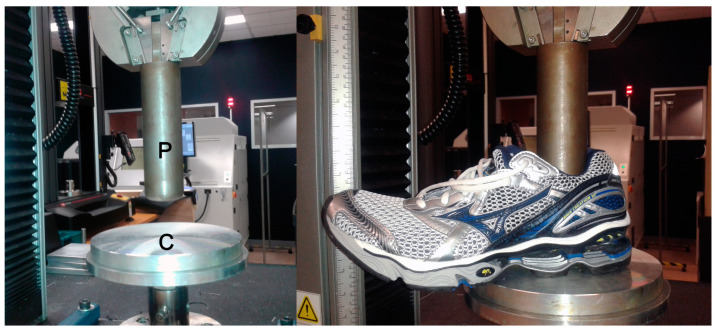
Test procedure for heel compression (Mizuno Wave Creation 12); P = plunger; C = compression platen.

The shoes were tested (Figure 5) using a modified ASTM-F1614-99 [8], Procedure B (force-controlled). The following modifications were required:

(1) ASTM-F1614-99 [8] specifies that a maximum energy in the range of 5 ± 0.5 J be applied to the shoes. Since the optimum point of energy absorption is not necessarily at or before the 5 J limit, the heel segment of the shoes was compressed up to 3 kN, and when the optimum point was not within this force range, larger compression forces up to 5 kN were applied. *Note 3* of ASTM-F1614-99 (*There is no evidence to support comparisons of data for tests which used … different reference maximum energy …* [8]) is not applicable because the shoulder point parameters remain the same regardless of the maximum compression force as long as it exceeds the optimum point. In contrast to this note, in light of the results of the present study, we consider a limit of 5 J as inappropriate, since the optimum point in terms of optimum displacement *x*, force *F*, and energy *E* varies from shoe to shoe.
(2) Anticipating the results of Section 2.3. (test for compression speed dependence of shoulder point parameters), the displacement profile used for heel compression was triangular (rise, fall, dwell), with a constant deflection rate of 10 mm/s up to the selected maximum force, programmed on static material testing machines (Instron 5569, Norwood, MA, USA; Z050, ZwickRoell GmbH & Co. KG, Ulm, Germany). The dwell segment resulted from the air gap between the plunger and the inner sole. The displacement profile was applied for 5 cycles, and the data from the last cycle were processed to calculate the shoulder point data. The data sampling frequency was 200 Hz.

This method was applied to 7 sub-studies to address specific aspects of this method, which are explained below.

### 2.6. Influence of Conditioning and the Minimum Number of Cycles Required for Testing

For viscoelastic solids, repeated loading cycles at small cycle numbers show a transient phase, e.g., decreasing peak forces, before reaching a steady state. This sub-study was designed to test whether the shoulder point parameters also undergo a transient phase and how many cycles are required to reach the steady state. A visual inspection of pre-test data showed that the steady state starts with the fifth cycle. To verify this result, six shoes (Asics GelKayano17, Mizuno WaveCreation 12, Nike Free, Brooks GTS, Adidas SpringBlade and Adidas MegaBounce) were tested. The four shoulder point parameters of each shoe were normalised to the reference value of cycle 5, and the following reciprocal function

(1) $$P={\left(a\;n-b\right)}^{-1}+c$$

was fitted to the data, where *P* denotes any shoulder point parameter, *a* is the amplitude parameter, *n* is the cycle number, *b* denotes the phase shift, and *c* is the offset. A difference of 0.01 (1%) between the normalised parameter at *n* = 4 predicted from Equation (1) and the normalised parameter at *n* = 5, which was equal to 1, was considered acceptable to identify cycle 5 as the beginning of steady state.

### 2.7. Repeatability of Shoulder Point Parameters

The six shoes used in the previous sub-study were each tested 10 times, with a rest period of at least 24 h between each test. Each shoulder point parameter was normalised to the mean of each shoe, expressed as a percentage minus 100%, i.e., the percentage difference between individual data and their mean. The uncertainty was calculated from the standard deviation of this difference. The standard error of the mean (=0%) was calculated from the uncertainty divided by √60.

### 2.8. Comparison of Shoulder Point Parameters of Different Shoes

The shoulder point parameters of all shoes were plotted against each other (e.g., *E*/*F*_max_ vs. *E*_opt_) to identify various design trends, outliers, and classification schemes (e.g., not or poorly designed for energy absorption, under-designed, well-designed, over-designed), and to determine how these trends and classifications relate to the leading athletic shoe companies. This graphical and interpretation-based approach is used to understand the dynamic interaction between shoulder point parameters and the design of a shoe heel. Since the six shoes in Cohort 3 represent a defined range from minimum to maximum shoes, we examined how the shoulder point parameters relate to, and vary within, this range.

The shoulder point parameters of the Cohort 3 shoes (3 sizes, left and right) were compared using the Kruskal–Wallis rank sum test for multiple independent samples, and the post–hoc Conover *p*-values of all possible pairs of the six groups were additionally adjusted using the Benjamini–Hochberg FDR method.

### 2.9. Influence of Shoe Size on Shoulder Point Parameters

If shoes of the same model but different size are tested with the same plunger size, we can assume that the magnitude of the shoulder point parameters depends on the shoe size *S* to some extent.

The following shoes and EU-sizes were available for this sub-study: Asics GelKayano17 (40.5, 47), Brooks GTS11 (40.5, 45.5), Mizuno WaveCreation12 (38.5, 46.5), Mizuno WaveElixir4 (39, 43), Mizuno WaveMusha3 (38.5, 42.5), Nike Free (40.5, 47), Joe Nimble Addict (39, 42, 45), Nike Free5.0 (40, 42.5, 45), Puma DeviateElite 2 (39, 42, 44.5), Puma Liberate (39, 42, 44.5), Puma Velocity2 (39, 42, 44.5), Vivobarefoot PrimusLite (40, 43, 46), Brooks Ghost15 (36.5, 40.5, 42). A total of 27 pairs (smaller and larger *S*) were tested, with a size difference, Δ*S*, between 1.5 and 8 (median 4, IQR 2.5). Shoes with 2 sizes counted as 1 pair, shoes with 3 sizes as 3 pairs. The calculated shoulder point parameters were compared using the Wilcoxon signed rank test (for correlated samples) to determine if the mean difference (between pairs) was equal to zero. To understand the effect of different sample areas, tested with the same plunger size, on the shoulder point parameters, samples of the same foam with areas of 25, 36, 49, 64, 81, and 100 cm^2^ and a thickness of 20 mm were tested using the same test procedure as described above (Figure 5), and the shoulder point parameters were calculated.

### 2.10. Influence of the Insole on the Shoulder Point Parameters

Sports shoes are usually equipped with a removable insole. The following available shoes were tested with and without an insole: Asics GelKayano17 (EU 40.5), Brooks Ghost15 (EU 36.5), Brooks Ghost15 (EU40.5), Brooks Ghost15 (EU42), Brooks GTS11 (EU 45.5), Mizuno WaveMusha3 (EU 41), Mizuno WaveRider12 (EU 43), Nike Free (EU 43). The calculated shoulder point parameters were compared using the Wilcoxon signed rank test (for correlated samples) to assess whether the mean difference (between shoes with and without an insole) is equal to zero.

### 2.11. Proposal for a Design Forgiveness Measure

**Figure 6:**
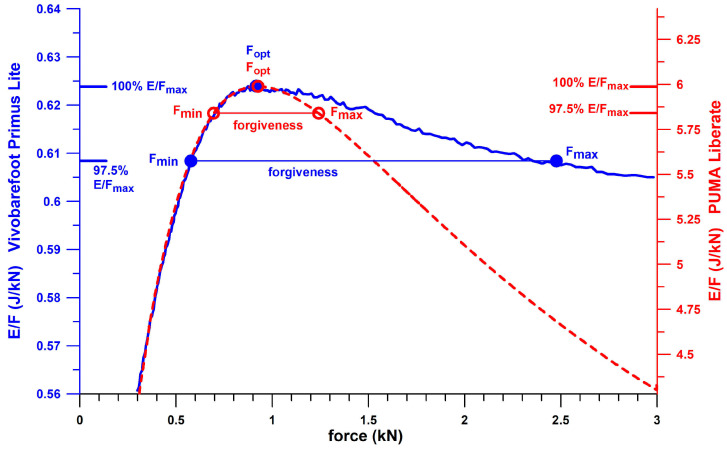
Forgiveness range of two shoes; ratio of energy E to force F versus force F; E/F_max_ = maximum ratio E/F at the shoulder point; F_opt_ = F at the shoulder point; F_min_, F_max_ = lower and upper boundary of the forgiveness range (= F_max_–F_min_).

Figure 6 shows the *E*/*F* vs. *F* curves of two shoes. The selected shoes, Vivobarefoot Primus Lite and PUMA Liberate, share the same *F*_opt_ (≈920 N), and the same ascending shank of the *E*/*F* vs. *F* curve, when plotting *E*/*F* with different scaling. A line at 97.5% of *E*/*F* vs. *F* intersects the *E*/*F* vs. *F* curve twice, before (*F*_min_) and after (*F*_max_) the shoulder point (peak). As explained in Section 2.2, heel soles are expected to feel softer when compressed to the shoulder point, compared to a harder feel well before or after the shoulder point. However, near the shoulder point, just before and after, when the *E*/*F* vs. *F* curve flattens out, there is probably no noticeable difference in hardness/softness. Therefore, if we reduce *E*/*F*_max_ by, say, 2.5%, and use the force window that is valid for 0.975 *E*/*F*_max_ ≤ *E*/*F* ≤ *E*/*F*_max_, that is, *F*_max_–*F*_min_, we get a measure of how compliant or forgiving the heel sole is designed to accommodate design errors. The forgiveness *ϝ* parameter is equal to the difference of *F*_max_–*F*_min_. A heel sole with a platykurtic shape of the *E*/*F* vs. *F* curve is more forgiving than that of a leptokurtic shape. Rather than using the actual kurtosis value of the *E*/*F* vs. *F* curve, a force window is more comprehensible and practically applicable. The reason for selecting the force window across and above a level of *E*/*F*_max_ minus 2.5% resulted from the problem that the heel soles had to be compressed to acquire force data covering the *F*_max_ reference point after the shoulder point. As shown in Figure 6, the PUMA Liberate shoe is less forgiving (*ϝ* = 546 N) than the Vivobarefoot shoe (*ϝ* = 1801 N) with more than three-times-larger *ϝ*. *F*_min_, *F*_max_, and *ϝ* were normalised to *F*_opt_, expressed as a percentage, to effectively compare shoes with different *F*_opt_. The forgiveness factors *ϝ*% of the following shoes (3 sizes, left and right) were compared using the Kruskal–Wallis rank sum test for multiple independent samples, and the post–hoc Conover *p*-values of all possible pairs of the seven groups were additionally adjusted using the Benjamini–Hochberg FDR method: Vivobarefoot PrimusLite, Joe Nimble Addict, Nike Free5.0, Puma Liberate, Puma Velocity2, Puma DeviateElite 2, Brooks Ghost15.

### 2.12. Measurement of the First Force Peak

The first force peak of the vertical ground reaction force, *PF*1, was measured when running over a force plate [27]. For this purpose, 19 female and 18 male runners were recruited (age 23.05 ± 1.99 yr and 26.50 ± 7.23 yr, respectively). The inclusion criteria were an age between 20 and 45 years and a minimum weekly running distance of 12 km. All participants gave their informed consent for inclusion in the study before participating in the study. The study was conducted in accordance with the Declaration of Helsinki and approved by the Ethics Committee of the University of Bayreuth (Approval-No. 23–041).

The participants were asked to run with six predefined shoe models ranging from minimalist to maximalist running shoes (Cohort 3, Section 2.4) as well as barefoot and with their personal running shoes. Ground reaction forces and centre of pressure (COP) were recorded using two 3D force plates (9287, Kistler Instruments AG, Winterthur, Switzerland). Each participant’s running speed was measured using two light barriers (Speedtrap 2, Brower Timing Systems, Draper, UT, USA) placed 4 m apart with the force plate in between.

After a five-minute warm-up with the subject’s personal running shoes, runs were performed in a random order with the eight footwear conditions. For each condition, the participant first ran for one minute each on a treadmill (stellar med, h/p/cosmos sports & medical GmbH, Nussdorf-Traunstein, Germany) at 80% and 100% of their individual training speed and at 10 kph. Subsequently, six valid runs over ground were recorded at the individual training speed and a running speed of 10 kph. Based on the data on the vertical ground reaction force, the magnitude of first peak force *PF*1 was determined and measured and finally compared with *F*_opt_ of the shoe models.

### 2.13. Summary of the Sub-Studies Described in the Methods Section

**Figure 7:**
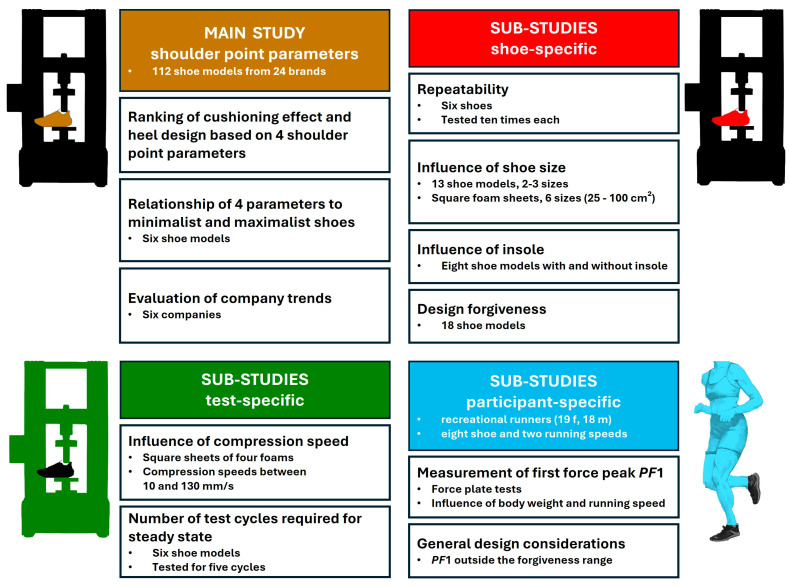
Flowchart of the study process; PF1: peak force 1, the first force peak of the Fz force.

Figure 7 summarises the various components of the study. It is divided into the main study, which directly addresses the evaluation of footwear using the shoulder point parameters, and three clusters of sub-studies addressing defined research questions necessary for a better understanding and practical applicability of the shoulder point parameters.

## 3. Results

### 3.1. Influence of Compression Velocity on Shoulder Point Parameters

**Figure 8:**
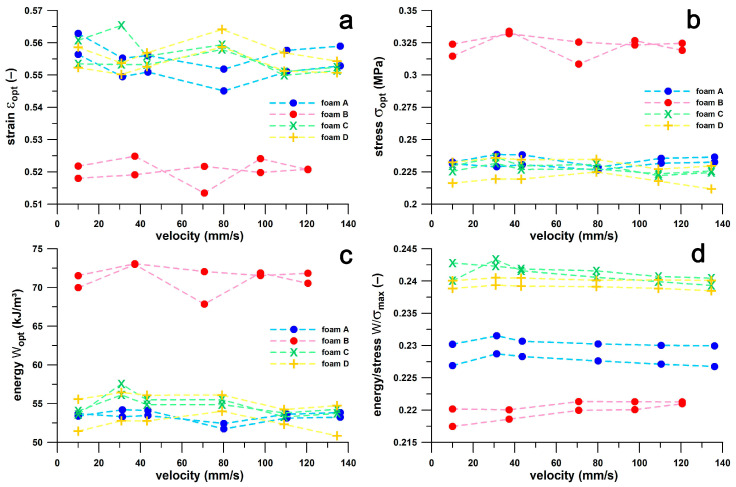
Shoulder point parameters of four foams (A, B, C, D; two samples each) versus crosshead speed (mm/s).

Figure 8 shows the four shoulder point parameters (maximum *W*/*σ*; and *ε*, *σ*, and *W* at the optimum) compared to compression speed. No clear velocity-dependent trend is evident. The Friedman rank sum test for correlated samples showed that no pair of velocity groups in *ε* and *σ* showed a velocity dependence (*p* = 0.423 and 0.107, respectively), while only one pair (out of 10) of *W*/*σ* and *W* showed a significant velocity dependence (*p* = 0.036 in both cases). The *W*/*σ* median (0.2349) at speed 2 (37–44 mm/s) was significantly larger than that (0.2342) at speed 5 (121–136 mm/s); and the *W*-median (55.2 kJ/m^3^) at speed 2 (37–44 mm/s) was significantly greater than that (53.7 kJ/m^3^) at speed 4 (98–110 mm/s). The significant velocity dependence in only two pairs compared to insignificant dependence in 38 pairs suggests that a static test is sufficiently accurate to calculate the four shoulder point parameters related to the optimal cushioning effect of sports shoes, and therefore confirms the hypothesis stated in Section 3.2.

### 3.2. Comparison of Shoes by Shoulder Point Parameters

**Figure 9:**
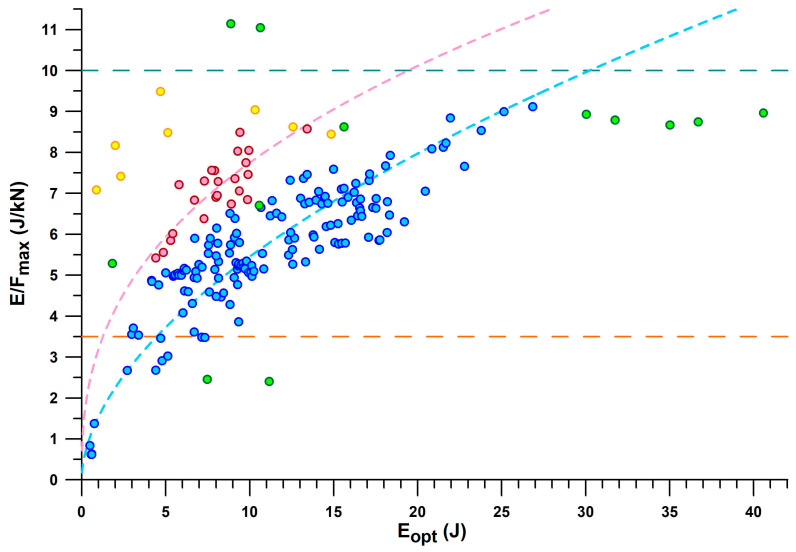
E/F_max_ versus E_opt_; blue dots: main line (cf. text of Section 3.2); red dots: secondary line; green dots: outliers (shoes that do not fit into main and secondary lines); yellow dots: additively manufactured shoe soles; dashed curves: power-law fits of main and secondary lines.

Figure 9 provides a comprehensive overview of all shoes by their *E*/*F*_max_ vs. *E*_opt_. The general principle of the main fit line (blue dots) and the secondary fit line (red dots) is that the more energy is absorbed, the greater the *E*/*F*_max_. For the same energy absorption between 4.5 and 13.5 J, *E*/*F*_max_ of the secondary line is 1.6–1.4 larger than that of the main line. The shoes located on the secondary line are mainly PUMA shoes (Puma_Deviate Elite 2, Puma_Liberate, Puma_Velocity 2, PumaFastR), as well as Brooks Ghost 11, CEP Omnispeed, On_Cloudneo (PEBAX foam), and Saucony_Endorphin.

Minimalist shoes are at the lower end of the *E*/*F*_max_ and *E*_opt_ scale, such as Vibram Bikla (“5 fingers”), Vivobarefoot, and Joe Nimble. The best shoe in the main line is the ASICS GelKayano17, with a maximum *E*/*F*_max_ of 9.1 J/kN. The best shoe on the secondary line is the CEP Omnispeed (prototype v5) shoe with a maximum *E*/*F*_max_ of 8.6 J/kN.

As can be seen in Figure 9, there are a few outliers. One Nike Airmax appears to be on the secondary line, but its cushioning technology is based on a pneumatic damper rather than closed-cell foam technology. Another Nike Airmax250 had its air bubble burst, so its *E*_opt_ was only 1.8 J, and its *F*_opt_ was only 0.35 kN, but its *E*/*F*_max_ was still an acceptable value of 5.29 J/kN compared to other shoes. The intact shoe of the same pair showed the following values: *E*_opt_: 10.6 J; *F*_opt_: 1.58 kN; and 6.71 J/kN.

**Figure 10:**
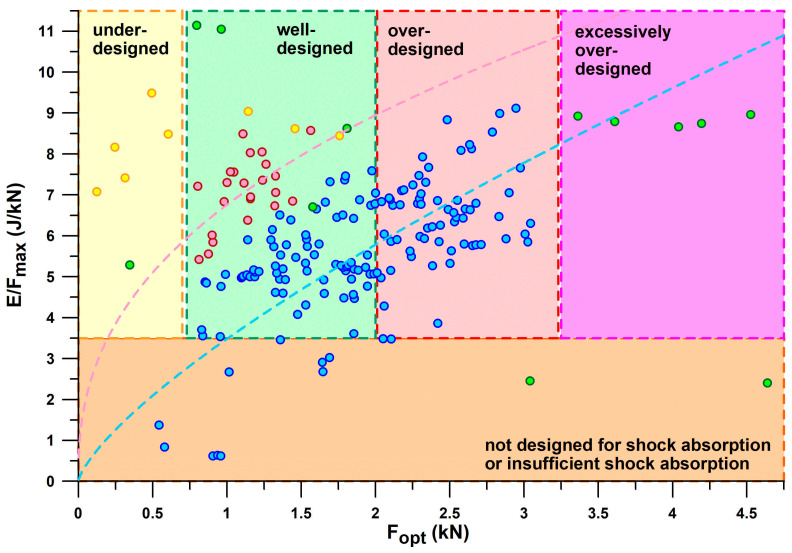
E/F_max_ versus E_opt_; blue dots: main line (cf. text of Section 3.2); red dots: secondary line; green dots: outliers; yellow dots: additively manufactured shoe soles; dashed curves: power-law fits of main and secondary lines.

Adidas Nitrocharge is an indoor soccer shoe with a thin sole and thus a very low *E*/*F*_max_ (2.4 J/kN) typical for minimalist shoes, but with a larger energy absorption than minimalist shoes. Nike ZoomAir is a skateboarding shoe with a stiffer heel segment. Adidas Bounce Tube shoes (MegaBounce and BounceTitan) absorb more energy (30.0–40.5 J) than other shoes with the same *E*/*F*_max_ ratio (8.8 J/kN on average), which is because the excessive *F*_opt_ ranges between 3.4 and 4.5 kN (Figure 10). A similar excessive *F*_opt_ is found in the Adidas Nitrocharge shoe with 4.6 kN, the largest *F*_opt_ force in the dataset, despite the smaller *E*_opt_ (11.2 J).

The largest *E*/*F*_max_ of the dataset is found in the PUMA RuleBreaker shoe, with 11.1 J/kN (presumably due to the increased sole thickness), at an *E*_opt_ of 8.9 J and 10.3 J (heel only, and heel + midsole pad, respectively) and an *F*_opt_ of 0.80 kN and 0.96 kN, respectively. The 3D-printed shoes consist of two batches characterised by *E*_opt_ < and > 7.5 J, due to smaller and thicker strut diameters. All 3D-printed shoes have a large *E*/*F*_max_ between 7 and 9.5 J/kN.

A cluster of shoes with *E*/*F*_max_ < 3.5 J/kN (Figure 9) and *x*_opt_ < 13.5 mm is considered as shoes with insufficient energy absorption. This cluster consists of the following shoes: Vivobarefoot_Primus Lite (minimalist shoe), Vibram Bikla (minimalist shoe), Bugatti (dress shoe), Adidas Nitrocharge (indoor soccer shoe), Nike ZoomAir (skateboard shoe), INov-8-roadx233, Nike Jordan1 Zoom (worn), Nike HyperVenom (soccer shoe), Adidas Fluidstreet (minimalist shoe, worn), Nike Total90 (soccer shoe), and Brooks Adrenalin. The insufficient energy absorption could have different reasons: (1) the shoes were not designed for energy absorption (soccer and skateboard shoes; minimalist shoes; dress shoes); (2) the shoes were worn regularly for more than one year; or (3) the low energy absorption is an inadvertent effect of the shoe design.

The shoes that were just outside the group of shoes with insufficient energy absorption were Joe Nimble Addict, Nike ZoomHyperfuse, and NewBalance (550 and 1150).

Figure 10 shows *E*/*F*_max_ versus *F*_opt_, taking the information provided in Figure 9 one step further. Based on the threshold of *E*/*F*_max_ < 3.5 J/kN to define shoes with insufficient energy absorption, and the window of the first peak force (*PF*1) of the vertical ground reaction force during running, the shoes can be further classified. *PF*1 ranged from 0.69–1.97 kN (1.17 ± 0.28 kN) during valid heel strikes of the participants studied. Based on these limits (0.7 and 2 kN), we can define a shoe range suitable for well-designed heel segments for running. Well-designed means that *PF*1 is close to *F*_opt_ within this range, depending on body weight, running motion (within the heel-striker cohort), and running speed. Shoes with Fopt < 0.7 kN are under-designed (first batch of 3D-printed shoe soles with thinner struts; also Nike Airmax 270 with a perforated bladder), which leads to running after the shoulder point, where energy absorber densifies and eventually maxes out. Heels with 2 kN < *F*_opt_ < 3.2 kN are over-designed, leading to running before the shoulder point and thus the runner cannot reach this optimum point. This means that runners run at *E*/*F* values smaller than the *E*/*F*_max_ value. Heels with *F*_opt_ > 3.2 kN are excessively over-designed, which only applies to Adidas Bounce Tube shoes. The classification of the shoes into under-, well-, and over-designed heel segments only applies to the group of participants studied (body mass: 68.4 ± 10.4 kg, range 47–91.8 kg), who were normal-weight recreational runners. As will be shown later in this document, runners with a higher body mass run with a higher *PF*1.

**Figure 11:**
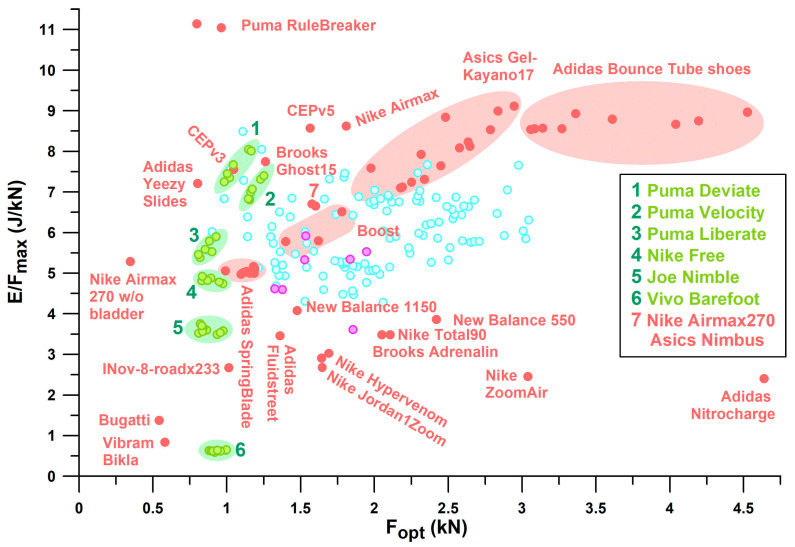
E/F_max_ versus E_opt_; identification of selected shoes; purple dots: basketball and handball shoes; blue dots: remaining shoes not further specified; Boost: thermoplastic polyurethane particle foam (one Puma shoe, two Adidas shoes).

If only shoes that are in the range of well-designed heel segments (0.7 kN < *F*_opt_ < 2.0 kN) are considered, the best-performing shoes of the major sports shoe manufacturers examined (Figure 11), from largest best to smallest best *E*/*F*_max_ per manufacturer, are as follows:

Puma: Rulebreaker, *E*/*F*_max_ = **11.05–11.14** (J/kN).

Nike: Airmax, *E*/*F*_max_ = **8.63** (J/kN).

Brooks: Ghost15, *E*/*F*_max_ = **7.75** (J/kN).

Mizuno: Wave Rider, *E*/*F*_max_ = **7.59** (J/kN) for *F*_opt_ < 2 kN. The largest *E*/*F*_max_ of WaveElixir4, namely 8.09, is not applicable, as the shoe is over-designed (*F*_opt_ > 2 kN), which means that runners cannot reach the shoulder point.

Adidas: Yeezy Slide at the lower limit of the *PF*1 range with an *E*/*F*_max_ of **7.21** (J/kN); the largest *E*/*F*_max_ of Bounce Tube shoes ranging from 8.54 to 8.97 (no other Adidas shoe within this range) is not applicable, as these shoes are excessively over-designed (*F*_opt_ > 3.2 kN).

Asics: Nimbus, *E*/*F*_max_ = **6.66**; Gel Kayano17 with the largest *E*/*F*_max_ of 7.1–9.12 (no other Asics shoe within this range) is not applicable as the shoe is over-designed (*F*_opt_ > 2 kN).

**Figure 12:**
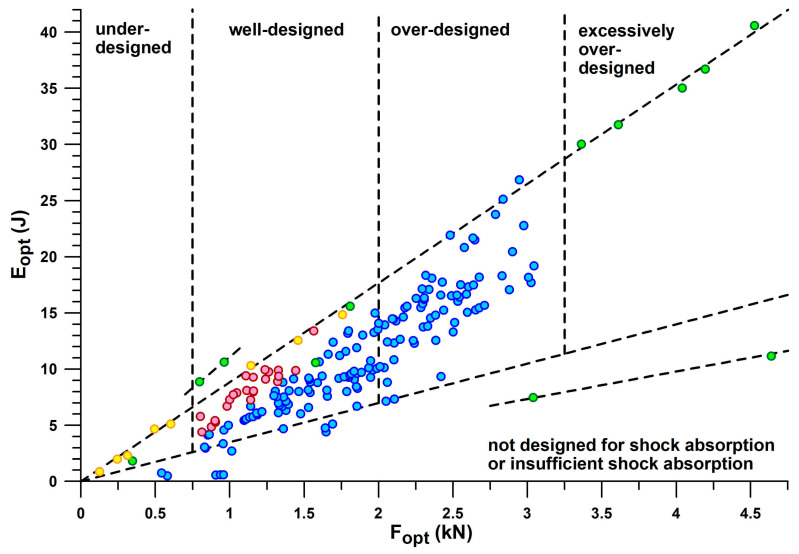
E_opt_ versus F_opt_; blue dots: main line; red dots: secondary line; green dots: outliers; yellow dots: additively manufactured shoe soles.

Figure 12 combines the information contained in Figure 10 and Figure 11 individually by plotting *E*_opt_ vs. *F*_opt_, which reflects the mechanical principle that the greater the force applied to a shock absorber, the more energy is absorbed. Any straight line through the origin of the graph shown in Figure 12 has a slope equal to *E*/*F*_max_. Shoes located on steeper lines have a better energy absorption at the heel sole. The advantage of Figure 12 is that it graphically reduces the *E*/*F*_max_-variation of 3D-printed soles, CEP Omnispeed (prototype 5), Nike Airmax, Asics Gel Kayano 17 and Adidas Bounce Tube shoes, so that they appear to lie on a line of *E*/*F*_max_ ≈ 8.834 J/kN.

**Figure 13:**
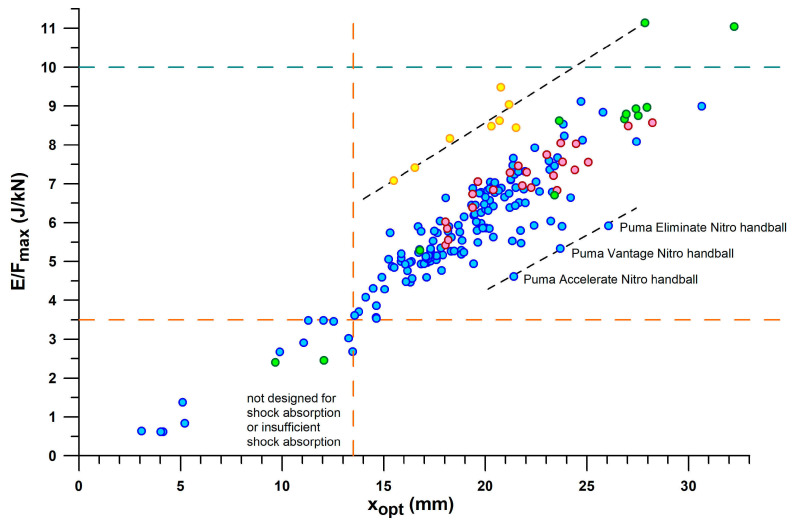
E/F_max_ versus x_opt_; identification of selected shoes; blue dots: main line; red dots: secondary line; green dots: outliers; yellow dots: additively manufactured shoe soles; the dashed lines show that three Puma shoes are located on one line at the lower end of the point cloud, and 3D-printed shoes plus the Puma Rulebreaker shoe (heel only) on another line at the upper end.

Figure 13 shows the plot of *E*/*F*_max_ vs. *x*_opt_. Any straight line through the origin of the graph (Figure 13) has a slope equal to *E*/*F*_max_/*x*_opt_. This diagram adds another dimension to the shoulder point parameters. While the shoes of main and secondary lines now overlap, other shoes are separated from the two lines, namely 3D-printed shoes with the largest ratio of *E*/*F*_max_ to *x*_opt_, which deflect less than other shoes with the same *E*/*F*_max_ (this also includes the Puma Rulebreaker shoe, heel only, without midsole pad). Another sequence of three shoes at the lower end of *E*/*F*_max_/*x*_opt_ have a common feature: they are all Puma handball shoes which deflect more than other shoes with the same *E*/*F*_max_. The separation of the cluster of shoes with inadequate energy absorption, defined above by *E*/*F*_max_ < 3.5 J/kN (Figure 9) *and x*_opt_ < 13.5 mm, is now clearly visible in Figure 13.

**Figure 14:**
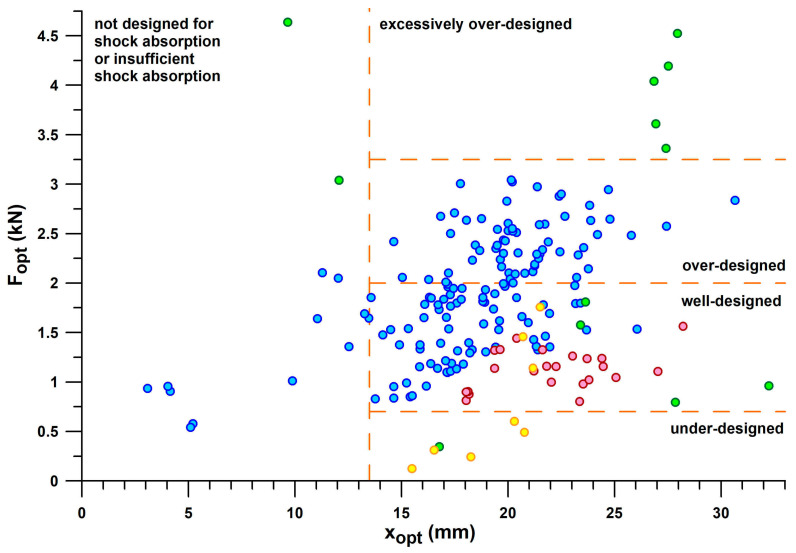
F_opt_ versus x_opt_; blue dots: main line; red dots: secondary line; green dots: outliers; yellow dots: additively manufactured shoe soles.

Figure 14 shows the plot of *F*_opt_ vs. *x*_opt_. This graph divides the shoes into clusters with insufficient energy absorption (*x*_opt_ < 13.5 mm) and the bulk of shoes, and the latter, in turn, into sections with under-designed, well-designed, and over-designed heel segments. It also shows that the maximum *x*_opt_ (>30 mm) is shared by two shoes, Puma Rulebreaker shoe (heel plus midsole pad) with *x*_opt_ of 32.2 mm, and Asics Gel Kayano 17 with *x*_opt_ of 30.7 mm. At approximately the same *x*_opt_, Asics Gel Kayano 17 produces an *F*_opt_ three times higher than the Puma Rulebreaker.

**Figure 15:**
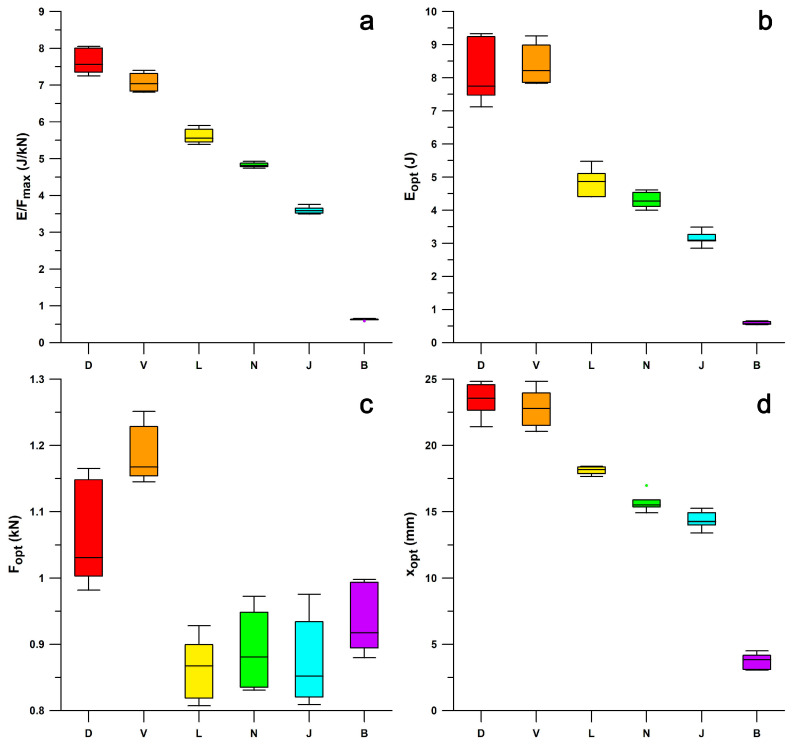
Shoulder point parameters of shoes of Cohort 3; boxplots of (**a**) E/F_max_, (**b**) E_opt_, (**c**) F_opt_, (**d**) x_opt_; ● = outlier; B = Vivobarefoot, J = Joe Nimble, N = Nike Free, L = Puma Liberate, V = Puma Velocity, D = Puma Deviate Elite.

Comparing minimalist shoes to maximalist shoes (Figure 15), it appears that *E*/*F*_max_ and *x*_opt_, and *E*_opt_ correlate well (R^2^ > 0.92) with the minimalist index and the stack height, but not with *F*_opt_ (R^2^ < 0.46). The minimalist index correlated well with the stack height (R^2^ = 0.9533). From the Kruskal–Wallis statistic and the post–hoc Conover *p*-values, *E*/*F*_max_ (Figure 15a) was significantly different in all six shoes (*p* < 0.0004). For *E*_opt_ (Figure 15b) there was no significant difference between Puma Velocity and Puma DeviateElite (*p* = 0.3228); all other shoes were significantly different (*p* < 0.0092). For *F*_opt_ (Figure 15c), there was no significant difference between Puma Velocity and Puma DeviateElite (*p* = 0.1899, although their IQRs only slightly overlapped), and the remaining four shoes (0.0588 < *p* < 0.7708). In both *E*_opt_ and *F*_opt_, the means and averages of Puma DeviateElite were smaller than those of Puma Velocity, although not significantly different. For *x*_opt_ (Figure 15d), there was no significant difference between Puma Velocity and Puma DeviateElite (*p* = 0.4112); all other shoes were significantly different (*p* < 0.0025).

**Figure 16:**
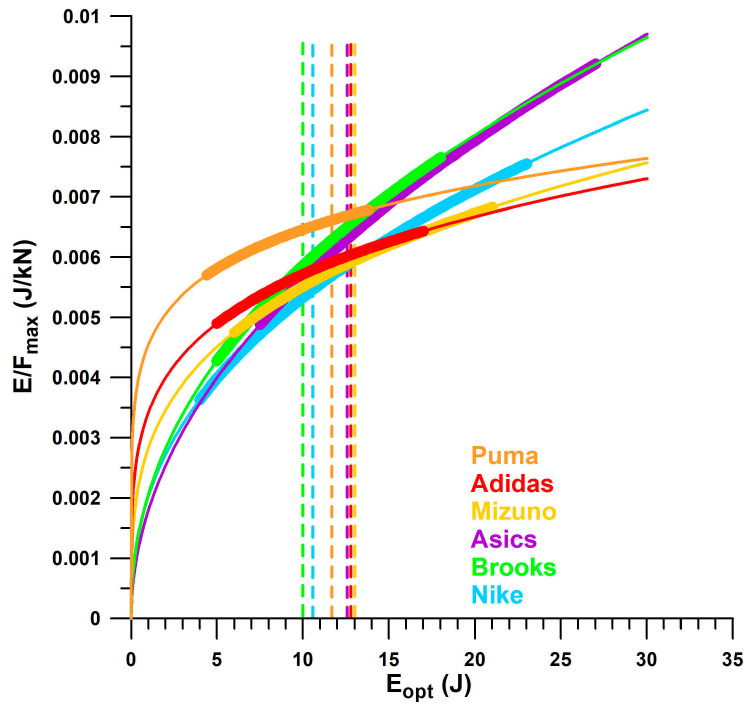
Company trends (refers only to the shoes tested in this publication; Puma shoes without Rulebreaker; Adidas shoes without Bounce Tube shoes); thin curves = general power law trend; bold curves = curve segment for the actual E_opt_ range; vertical dashed lines = energy value approximately corresponding to F_opt_ at 2 kN.

Of the six major companies identified in Table 1, Asics and Brooks shoes show the same trend of *E*/*F*_max_ vs. *E*_opt_, while Nike shoes show a slightly smaller *E*/*F*_max_ with a larger *E*_opt_ (Figure 16). Likewise, Puma, Adidas, and Mizuno share the same trend, with Puma shoes showing on average a 10% to 20% higher *E*/*F*_max_ than Adidas and Mizuno, between 5 J ≤ *E*_opt_ ≤ 15 J. Vertical lines mark the beginning of over-designed heels, i.e., from *F*_opt_ ≈ 2 kN. Puma shoes had the smallest *E*_opt_ range within the window of 2 kN ≤ *F*_opt_ ≤ 3 kN, while Asics had the widest (i.e., maximum number of over-designed shoes). Of all the shoe models tested across the six major companies, Asics shoes had the lowest number of well-designed heel soles (54%), while Puma had the highest number (93%). In addition, Puma shoes have, on average, the largest *E*/*F*_max_ value within the well-designed range.

### 3.3. Transient Phase and Steady State of Shoulder Point Parameters with Respect to the Test Cycle Numbers

**Figure 17:**
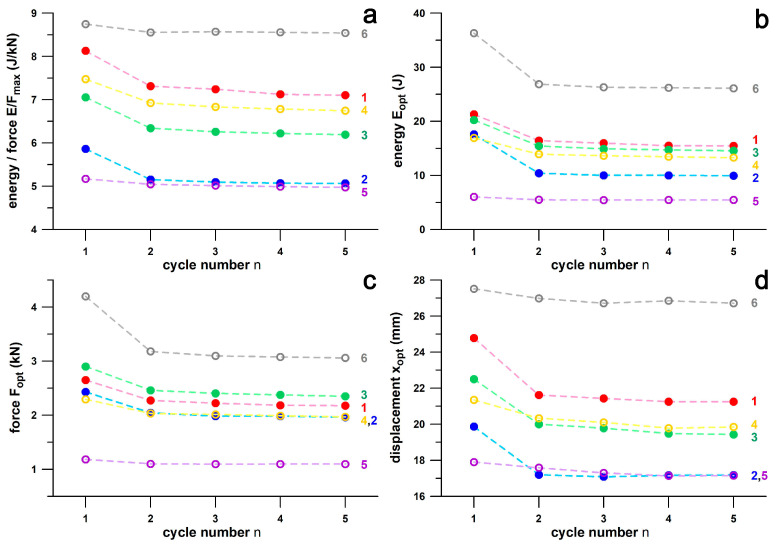
Shoulder point parameters vs. cycle number; (**a**) energy/force; (**b**) energy; (**c**) force; (**d**) displacement; 1: Asics GelKayano17, 2: Mizuno WaveCreation12, 3: Nike Free, 4: Brooks GTS, 5: Adidas SpringBlade, 6: Adidas MegaBounce.

**Figure 18:**
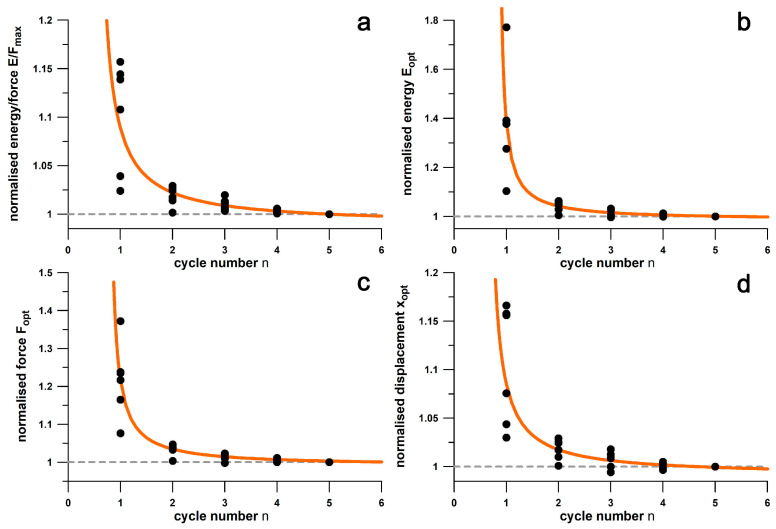
Normalised shoulder point parameters vs. cycle number; (**a**) normalised energy/force; (**b**) normalised energy; (**c**) normalised force; (**d**) normalised displacement; E: energy; F: force; x: displacement; ●: data of six shoes (cf. Figure 17); orange curve: fit function according to Equation (1).

Figure 17 shows the shoulder point parameters versus cycle numbers from 1 to 5. The data are generally larger in the first cycle. Figure 18 shows the data from Figure 17 normalised to the data of the fifth cycle and the fit function according to Equation (1). The differentials between the normalised parameter at *n* = 4 predicted from Equation (1) and the normalised parameter at *n* = 5 (at unity) were generally less than 0.01, and specifically 0.0032, 0.0051, 0.0040, and 0.0025 for *E*/*F*, *E*, *F*, and *x*, respectively. These results are less than 1% of the parameter size in the fifth cycle.

### 3.4. Repeatability Results of Shoulder Point Parameters

**Figure 19:**
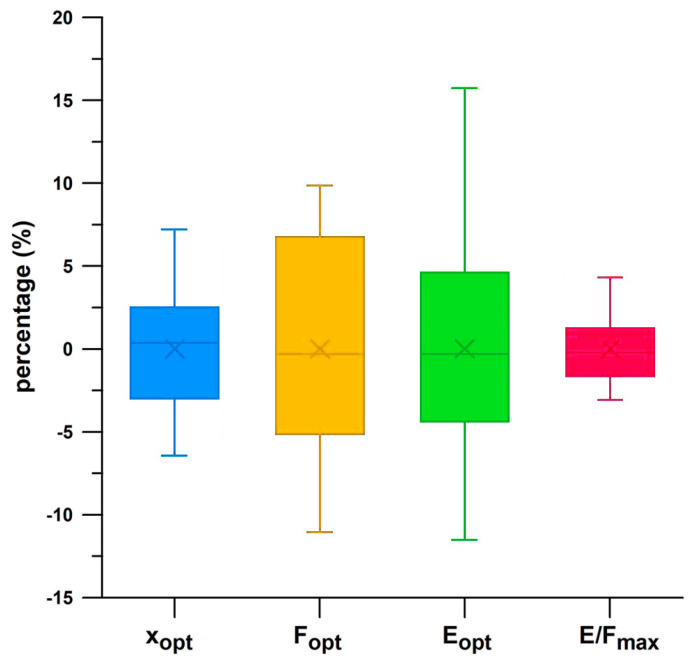
Repeatability of shoulder point parameters from six shoes combined, expressed as the percent deviation from the mean of each shoe.

Figure 19 shows the data distribution of ten tests per shoe about the mean of six shoes (percent deviation from the mean of each shoe). The uncertainty of *x*_opt_, *F*_opt_, *E*_opt_, and *E*/*F*_max_ was 3.24%, 6.01%, 6.32%, and 1.89%, respectively. The standard error of the mean of *x*_opt_, *F*_opt_, *E*_opt_, and *E*/*F*_max_ was 0.418%, 0.776%, 0.816%, and 0.244%, respectively. Table 2 shows the individual repeatability of the six shoes. Nike Free had the best repeatability on average, while Adidas MegaBounce had the worst.

### 3.5. Influence of the Shoe Size on Shoulder Point Parameters

The difference in shoulder point parameters between two shoes of sizes *S* was calculated from the differential between magnitude of larger *S* parameter and magnitude of smaller *S* parameter. The results of the Wilcoxon test showed that there were significant differences in all four shoulder point parameters (between two shoe sizes). For a mean Δ*S* of 4.15 (*p* < 0.0001), the mean of Δ*E*/*F* was 0.29 J/kN (*p* = 0.0003), the mean of Δ*E* was 1.21 J (*p* = 0.0002), the mean of Δ*F* was 0.11 kN (*p* = 0.0018), and the mean of Δ*x* was 1.13 mm (*p* = 0.0027). Note that all means were positive, which explains that larger shoe sizes correlate with larger *E*/*F*, *E*, *F*, and *x*. This means that when testing shoes with the same plunger dimensions, the shoulder point parameters of larger shoe sizes are overestimated compared to smaller sizes, or parameters of smaller shoe sizes are underestimated compared to larger sizes.

Across a mean Δ*S* of 4.15, which corresponds to 8.8% of the maximum size of *S*, i.e., 47, the changes in the shoulder point parameters are relatively small (after excluding Adidas Bounce Tube and Nitrocharge shoes with excessive *F*_opt_):

- Δ*F* of 0.11 kN corresponds to 3.6% of maximum *F*_opt_ (3.044 kN);
- Δ*E* of 1.21 J corresponds to 4.5% of maximum *E*_opt_ (26.85 J);
- Δ*x* of 1.13 mm corresponds to 3.5% of maximum *x*_opt_ (32.25 mm);
- Δ*E*/*F* of 0.29 J/kN corresponds to 2.6% of maximum *E*/*F*_max_ (11.14 J/kN).

**Figure 20:**
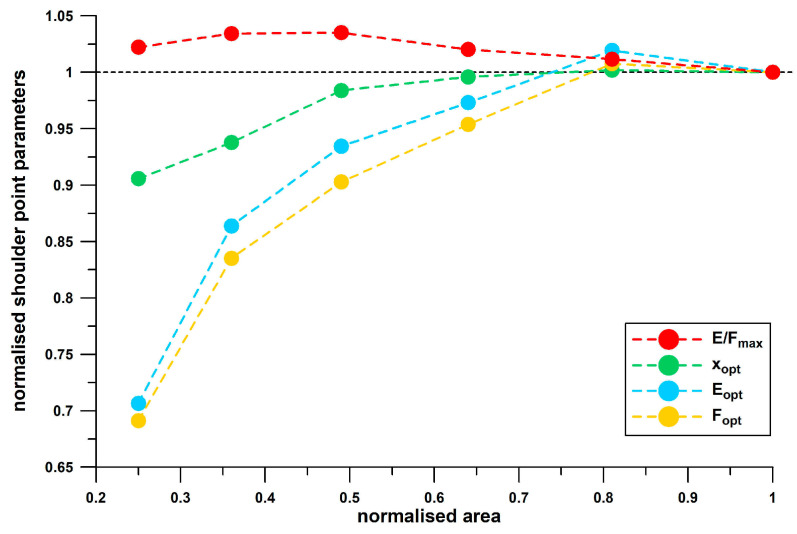
Normalised shoulder point parameters versus normalised area of foam sheets (100 cm^2^ corresponds to 1).

To understand the dynamics of testing different shoe sizes, square sheet samples of the same foam material but with varying side length were tested with the same plunger size (50 mm in diameter). Table 3 lists the sample area and the corresponding values of the shoulder point parameters. In Figure 20, the data are normalised to the values obtained at maximum area.

Figure 20 shows that the normalised shoulder point parameters, *x*_opt_, *F*_opt_ and *E*_opt_, increase nonlinearly with area, until the second largest area, where the data appear to asymptotically approach unity. This behaviour confirms the result obtained from the heel soles. Since the normalised *E*_opt_ data are slightly larger than the *F*_opt_ data, the normalised *E*/*F*_max_ values are greater than 1 and decrease with increasing area, and asymptotically approach 1 starting at the second largest area. This behaviour contrasts with the result obtained from the heel soles, where *E*/*F*_max_ increases with increasing size.

### 3.6. Influence of the Insole on Shoulder Point Parameters

The difference in the shoulder point parameters between two pairs was calculated as follows: the magnitude of the shoe parameter with insole minus magnitude of the shoe parameter without insole. The results of the Wilcoxon test showed that there were significant differences in all four shoulder point parameters (between with and without insoles). The mean of Δ*E*/*F* was 0.65 J/kN (*p* = 0.0005), the mean of Δ*E* was 0.67 J (*p* = 0.0271), the mean of Δ*F* was –49.3 N (*p* = 0.0308), and the mean of Δ*x* was 4.17 mm (*p* = 0.0005). While the means of Δ*E*/*F*, Δ*E*, and Δ*x* were larger than zero, the mean of Δ*F* was slightly but still significantly less than zero.

### 3.7. Design Forgiveness Parameter

**Figure 21:**
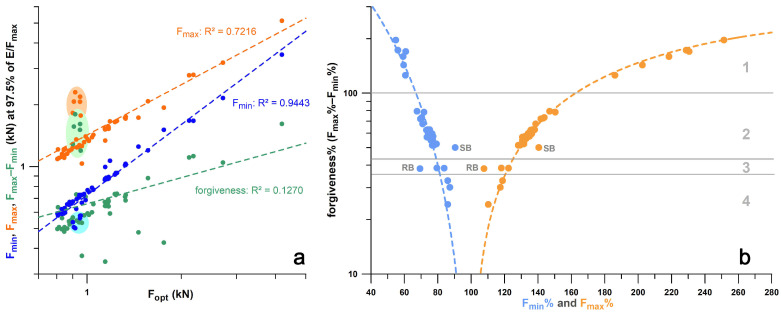
Correlations of force markers (cf. Figure 5) at E/F > 0.975 E/F_max_: (**a**) minimum (min) and maximum (max) forces and their differential (forgiveness) vs. F_opt_; the ellipses shaded in light orange; green and blue indicate the outliers (Vivobarefoot Primus Lite); (**b**) forgiveness as percentage of F_opt_ vs. F_min_ and F_max_ as percentages of F_opt_; the second-order regression functions (dashed) converge to 100% at a forgiveness at 0 N; SB = Adidas SpringBlade; RB = PUMA RuleBreaker (heel only); zones 1,2,3,4 categorise different clusters of shoes; zone 1: Vivobarefoot Primus Lite; zone 2: main bulk of shoes (PUMA: Deviate, Liberate, Velocity, FastR, RuleBreaker with midsole; Joe Nimble Addict; Nike Free; Gel Kayano; Nike Airmax; CEP Omnispeed Bowtech Bounce; Saucony Endorphin; Brooks Ghost); zone 3: PUMA RuleBreaker (heel only), NikeAirVisiPro, Adidas MegaBounce; zone 4: 3D-printed shoe soles.

In general, the larger *F*_opt_, the larger the *F*_min_, *F*_max_, and forgiveness *ϝ* (Figure 21a). However, the R^2^ value of *F*_opt_ vs. *ϝ* is only 0.1270 (*p* = 0.0047). The correlations shown in Figure 21a are influenced by a cluster of outliers (highlighted with coloured ellipses in Figure 21a). These outliers are exclusively the Vivobarefoot Primus Lite shoes, with an excessive *ϝ* between 1 and 2 kN, a range expected by the linear regression function at 0.6 kN. When the Vivobarefoot outliers are excluded, R^2^ improves to 0.7478. The same outlier problem occurs when plotting *ϝ* vs. *F*_min_ and *F*_max_. However, when plotting the normalised parameters *ϝ*% against *F*_min_% and *F*_max_% (Figure 21b), non-linear regressions (second-order polynomial) reduce the outliers to a minimum. The normalised forgiveness *ϝ*% of Vivobarefoot Primus Lite shoes ranges from 100% to 200%, while *ϝ*% of most of the other shoes range between 45% and 85% (Figure 21b).

**Figure 22:**
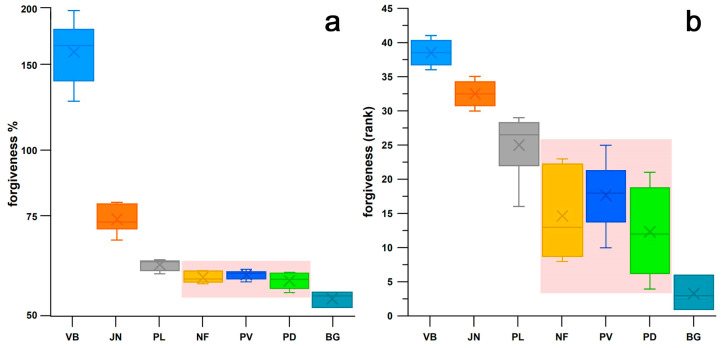
Box-and-whisker plots of the forgiveness factor ((**a**) as a percentage of *F*_opt_, and (**b**) as rank numbers); the area shaded in pink indicates that forgiveness factor of these three shoes is not significantly different; VB: Vivobarefoot PrimusLite, JN: Joe Nimble Addict, NF: Nike Free5.0, PL: Puma Liberate, PV: Puma Velocity2, PD: Puma DeviateElite 2, BG: Brooks Ghost15.

**Figure 23:**
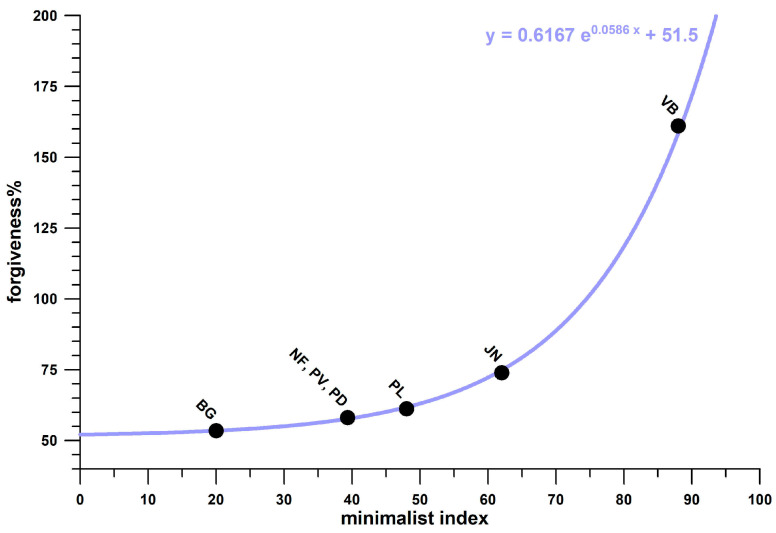
Normalised and averaged forgiveness factors (as a percentage of *F*_opt_) vs. minimalist index (index reported by [27]); VB: Vivobarefoot PrimusLite, JN: Joe Nimble Addict, NF: Nike Free5.0, PL: Puma Liberate, PV: Puma Velocity2, PD: Puma DeviateElite 2, BG: Brooks Ghost15; NF, PV, and PD are considered as a single group since their forgiveness medians are not significantly different (cf. Figure 22).

Figure 22 shows the boxplots of the actual values and the corresponding ranks of seven groups of shoes (cohort 3, and Brooks shoes of cohort 4). The ranks were used to calculate the Kruskal–Wallis statistic. From the post–hoc Conover *p*-values, it was found that there was no significant difference between three shoes (Figure 22): Nike Free, Puma Velocity, and Puma DeviateElite. Averaging the *ϝ*% data of these three groups as well as the corresponding minimalist indices *MI* [27], and adding the *ϝ*% data and *MI* of the remaining four significantly different groups, reveals that *ϝ*% appears to be an exponential function of *MI* (Figure 23).

### 3.8. First Peak of the Vertical Ground Reaction Force and Its Relationship to the Forgiveness Range

**Figure 24:**
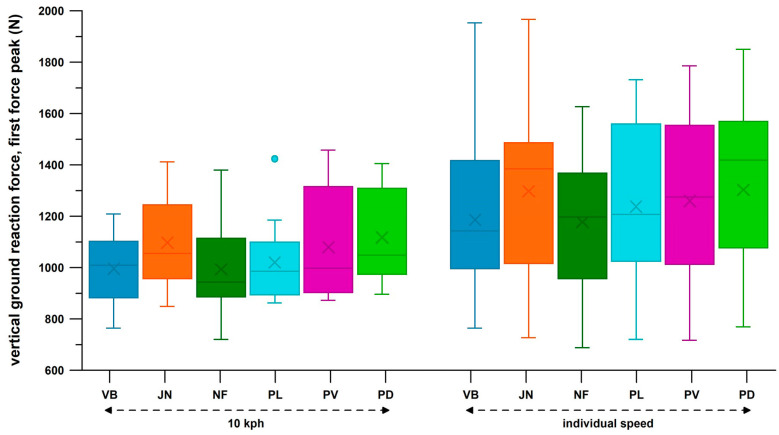
Box-and-whisker plot of the first force peak of the vertical ground reaction force across six shoes and two running velocities; VB: Vivobarefoot PrimusLite, JN: Joe Nimble Addict, NF: Nike Free5.0, PL: Puma Liberate, PV: Puma Velocity2, PD: Puma DeviateElite 2.

Figure 24 shows the distribution of the first peak force, *PF*1, across six shoes and two velocity groups of heel strikers. The medians of *PF*1 when participants ran at 10 kph were generally smaller than those when running at individual speed (9.3–13.3 kph). For both speed groups, the significantly different pairs were (Friedman test, and post–hoc Dunn–Bonferroni test): 10 kph: the medians of Vivobarefoot and Nike Free were significantly smaller than those of Puma Deviate (both at *p* = 0.017); individual speed: the median of Vivobarefoot was significantly smaller than that of Joe Nimble (*p* = 0.047); and the median of Nike Free was significantly smaller than those of Joe Nimble (*p* < 0.001) and Puma Deviate (*p* = 0.001). All other pairs showed no significant difference.

When plotting *PF*1 with respect to the actual forgiveness range, almost all cases of 10 participants with *PF*1 across all experiments were within the forgiveness range (Table 4) when running at 10 kph. The exceptions were Joe Nimble Addict, with four cases of *PF*1 larger than *F*_max_, and Puma Liberate, with two cases. This result generally indicates that the participants were running at the shoulder point, or at least within the forgiveness range. While this study suggests that the shoulder point represents the optimum point of energy absorption, a parameter related to shoe design, this result provides the evidence that running at the shoulder point actually occurs regardless of the *E*/*F*_max_ ratio.

**Figure 25:**
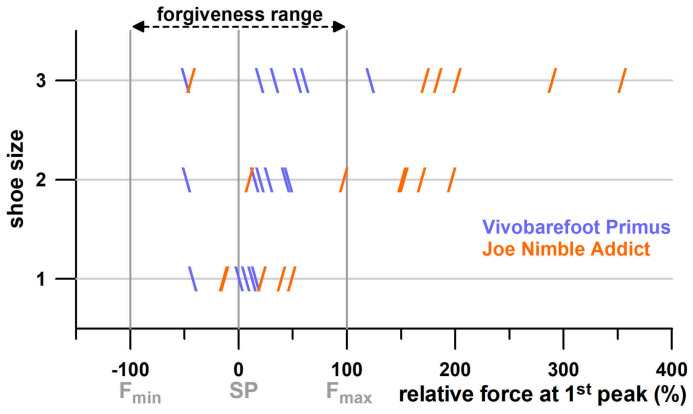
Size of two shoes, run at individual speed, against the position of the force at the first peak (PF1) relative to the shoulder point and the forgiveness range; F_min_ (beginning of forgiveness range) is set to −100%; and F_max_ (end of forgiveness range) is set to +100%; SP = shoulder point; shoe sizes of 1, 2, and 3 correspond to small, medium, and large sizes.

When running at the individual speed, the *PF*1 distribution changed dramatically (Table 4). *PF*1 larger than F_max_ was found in 17 participants across all experiments. Apart from three cases with *PF*1 smaller than *F*_min_, only two shoes had a minimum number of cases of *PF*1 larger than *F*_max_, namely Vivobarefoot, with one case (Figure 25), and Puma Velocity, with two cases. For the remaining four shoes, approximately 45% of cases were inside the forgiveness range, and 55% were outside, larger than *F*_max_ (Figure 25). This result shows that four shoes (Table 4) are partially under-designed, specifically when running at individual speed and also when running at a higher *BW* (Table 5). In contrast to Figure 10, which does not identify under-designed commercially available sports shoes across a wide range of *PF*1 values, focussing on *PF*1 data of individuals (participants of this study) reveals potentially under-designed shoes under certain conditions.

For all six shoes, the 39 cases where the shoes were larger than *F*_max_ were distributed among shoe sizes small, medium, and large, with 1, 17, and 21 cases, respectively. This result suggests that shoe size *S* and/or body weight *BW* influence how many cases were outside the forgiveness range. Multiple regressions of two predictors, *S* and *BW*, and dependent variable of *PF*1, revealed that, at a speed of 10 kph and at individual speed, the shared components (combined influence of *S* and *BM* on *PF*1) were 22.33% and 19.24%, respectively; the unique influences of *BW* on *PF*1 were 20.19% and 16.32%, respectively; and the unique influences of *S* on *PF*1 were 0.11% and 0.89%, respectively. The negligible unique influence of *S* on *PF*1 indicates that *BW* generally contributes the dominant influence.

To address the issue of why larger sizes produce outliers with *PF*1 larger than *F*_max_ (Table 4 and Figure 25), the following question arises: which parameter is significantly different between *PF*1 outliers and *PF*1 data smaller than *F*_max_, and, apart from the magnitude of *PF*1, which parameter was the larger *PF*1 associated with? When dividing the *PF*1 data into two groups, based on *PF*1 larger or smaller than *F*_max_, and comparing the associated medians of *BW*, body height *BH*, shoe size *S* and running speed *v* with the Mann–Whitney U test, all medians were significantly different. *PF*1 > *F*_max_ was associated with significantly larger medians of *BW*, *BH*, body mass index, *S*, and *v* (Table 5). Equally, the gender distribution was significantly different between the two groups, with 44% and 0% of female participants in the groups with *PF*1 < *F*_max_ and *PF*1 > *F*_max_, respectively (Table 5).

That the medians of the two *PF*1 groups were significantly different is evident, since *PF*1 (smaller or greater *F*_max_) was the decision criterion to separate the two groups. Of the remaining two large effect sizes (Table 5), *BW* has a larger effect on *PF*1.

According to Table 6, the average *BW*, *BH*, *PF*1, and individual velocity increase significantly from small to medium and medium to large sizes (*p* < 0.002, Kruskal–Wallis test). The increase in *PF*1 relative to size and across the two speeds (10 kph and individual speed) is larger than that of *BW* and *BH*. This result reflects that shown in Figure 25, namely, that the change in individual speed relative to the three shoe sizes is responsible for the increased number of outliers, as shown in Table 4, and not *BW* or *BH*.

These results can be interpreted in two opposite but complementary ways:

(1) The larger shoe sizes were not designed for heavy runners;
(2) The participants were too heavy for larger shoe sizes.

Both interpretations are not applicable, as the increased running velocity relative to the three shoe sizes accounts for the increased number of outliers, as shown in Table 4. The alternative interpretation would be as follows:

(1) The larger shoe size design cannot keep up with the faster running speed in addition to the increased *BW*;
(2) The participants were too fast for current larger shoe size design.

### 3.9. Design Considerations and Strategies for Adapting Shoes to the Shoulder Point Parameters

At this point, the question arises: which design feature should be responsible for a larger *PF*1 in general and the larger *PF*1 due to the faster running speed?

The basic design features of energy absorbers are [14]:

(1) The design space: for sports shoes, this is the standardised length (shoe size in relation to the foot size [28], the width, and the thickness of the shoe sole; the thickness is limited to a maximum of 40 mm in road events (running and race-walking competitions [29].
(2) The peak deceleration (−*a*_peak_).
(3) The absorber properties, specifically, *ε*_opt_, *σ*_opt_, *W*_opt_, and *W*/*σ*_max_ (cf. Section 2.2.) and their non-normalised counterparts of *x*_opt_, *F*_opt_, *E*_opt_, and *E*/*F*_max_.

Using the non-normalised properties of the Puma DeviateElite shoe (*x*_opt_ = 0.0234 m, *F*_opt_ = 1.06 kN, *E*_opt_ = 8.10 J, *E*/*F*_max_ = 7.64 J/kN) and the corresponding design space parameters (stack height *d* = 0.038 m, approximate heel area *A* = 0.005 m^2^, and resulting heel volume *V* = 0.00019 m^3^), the normalised absorber properties are *ε*_opt_ = *x*_opt_/*d* = 0.6166 (-), *σ*_opt_ = *F*_opt_/*A* = 212000 Pa, *W*_opt_ = *E*_opt_/*V* = 42631.6 J/m^3^, and *W*/*σ*_max_ = 0.2011 (-). Assuming an effective mass *m* of 50 kg that must be decelerated, this results in −*a*_peak_ = −*F*_opt_/*m* = 21.2 m/s^2^. If the normalised absorber properties are assumed to be the standard of the heel material, new non-normalised properties of the heel structure can be calculated if the design space parameters are changed.

The usual method for designing energy-absorbing structures is to calculate the required design thickness of an energy-absorbing material and compare the calculated design thickness with the available design space. Accordingly, we increase the design thickness *d* by 15% (scaling factor), i.e., to *d* = 0.0437 m. The heel area remains unchanged (0.005 m^2^). The absorber volume increases to 0.0002185 m^3^. As a result, the non-normalised properties change as follows: *x*_opt_ = **0.0269** m, *F*_opt_ = 1.06 kN, *E*_opt_ = **9.32** J, and *E*/*F*_max_ = **8.79** J/kN (Table 7). Comparing these data to the original non-normalised properties, we find that all parameters have increased (improved) except one, namely, *F*_opt_. However, it is the force (*PF*1) that increases with increasing shoe size (due to the *BW* and speed; Table 6). This design result explains that thickening the heel sole has no effect. Evidently, as the force (*PF*1) increases, we also want to improve the energy absorption, which is what actually happened (from 8.10 J to 9.32 J), but the force was not affected. If the force does not keep up with *BW* and speed, then the shoe becomes under-designed and bottoms out.

Since the increase in stack height had no effect on the force, the next design step is to change the design area *A* by 15% in both dimensions, i.e., to *A* = 0.0066 m. The stack height *d* remains unchanged (0.005 m^2^). The absorber volume increases to 0.0002508 m^3^. The unnormalised properties then change to *x*_opt_ = 0.0234 m, *F*_opt_ = **1.40** kN, *E*_opt_ = **10.692** J, and *E*/*F*_max_ = 7.64 J/kN (Table 7). Comparing these data with the original non-normalised properties, an improvement in *F*_opt_ and *E*_opt_ by 32% (or by a factor of 1.32 = 1.15^2^) is obtained. As the force increases, the effective mass *m* that must be decelerated also increases: *m* = *F*_opt_/*a*_peak_ = 66 kg (increase of 32%). By increasing the design area, *F*_opt_ has been successfully improved to accommodate the increased *PF*1 and *BW* values in larger shoe sizes—in principle. The limitations of this approach are three-fold:

(a) If we increase the size of an object by a scaling factor, the volume of the object is equal to the scaling factor to the power of three. Since the weight is proportional to volume, we expect an improvement of 1.52 (= 1.15^3^) rather than 1.32 (= 1.15^2^). If we correlate the participants’ *BW* and *BH* data, we obtain a power-law regression function of *BW* = 116.49 *BH*^3.1403^ (R^2^ = 0.5208), where the exponent of 3.1 corresponds to the expected exponent (3) of the scaling factor. Thickening the stack height in addition to the already increased area does not solve the problem since the stack height does not change the force. Using an area of 1.15 times the shoe length and 1.32 times the shoe width would solve the problem, with the disadvantage that the width would then increase disproportionately with the scaling factor to the power of two.

(b) We have not yet taken into account the increase in running speed. Since the running distance and speed are unidirectional, and, therefore, we assume an increase in speed and deceleration of the effective mass by the same scaling factor (15%), then the new −*a*_peak_ = 24.38 m/s^2^. The new effective mass *m* to be decelerated is *m* = *F*_opt_/*a*_peak_ = 1.4 kN/24.38 m/s^2^ = 57.39 kg. This is an increase of only 15%. If we consider that the running speed increases by a factor of 1.077 relative to the increase in *BW*, based on the participants’ data, we need a scaling factor of 1.42 (1.32·1.077) for velocity and acceleration. The new effective mass *m* to be decelerated is *m* = *F*_opt_/*a*_peak_ = 1.4 kN/(21.2·1.077) = 61.28 kg. This is an increase of only 22.6%. Another approach is to derive the scaling factor of the speed from the principle of geometrical similarity. If two geometrically similar objects with different scaling factors *L* move with the same Froude number *Fr*, then their velocities *v* are calculated from *v*^2^ = *Fr* g *L*, where g is the gravitational acceleration [30,31,32]. Thus, *v* ∝ √*L*. Therefore, the new effective mass *m* that must be decelerated is *m* = *F*_opt_/*a*_peak_ = 1.4 kN/(21.2·1.15^0.5^) = 61.54 kg. This is the same increase of only 22.6%. At least this result provides a good agreement of theory (principle of geometric similarity) and experimental data.

(c) If we consider that the scaling factor of the originally assumed effective mass m of 50 kg is 1.15^3^, i.e., 1.52, then the new mass that must be decelerated by a force of 1.4 kN is 76 kg. Thus, the required deceleration −*a*_peak_ = −*F*_opt_/*m* = 1.4 kN/76 kg = 18.41 m/s^2^. This means that the original deceleration of 21.2 m/s^2^ is reduced by 13.2%, which is counterintuitive, since higher velocities require larger decelerations that must be reduced to zero under the same constraints (same stack height). There is another aspect of designing an energy absorber which helps verify this counterintuitive result. The velocity addressed before is a direct result of the absorbed energy and the effective mass: *v*^2^ = 2 *E*_opt_/*m*. Using the original data, √(2·8.1/50) = 0.569 m/s, the initial speed at which the energy absorber (heel sole) is compressed. Using the new data, √(2·10.692/76) = 0.530 m/s, results in a reduction by 6.85%. Since we obtain the same counterintuitive result, the only option left is to increase the area disproportionately to achieve an adequate *F*_opt_, such that *PF*1 is not larger than *F*_opt_. This was already suggested under a) above. This solution illustrates the dilemma of contemporary shoe design, namely that the width of the shoe sole does not increase more than the length or the shoe size, which is a function of the foot length *l_F_*: size EUR = 3 *l_F_*/20 + 2 [28].

From the outliers in Table 4 (*PF*1 > *F*_max_ at individual speed), we can calculate the required increase in heel area (expressed as increase in heel width, not heel length, since heel length is a function of shoe length) to have the *PF*1 data at the upper boundary of the forgiveness range (*F*_max_) or even at the shoulder point (*F*_opt_). Since

(2) $$\sigma_{opt}=\frac{F_{opt}}{A_{s}}=\frac{PF1}{A_{n}}$$

the area increase is given as

(3) $$\frac{A_{n}}{A_{s}}=\frac{PF1}{F_{opt}}$$

where *A*_s_ is the standard heel area of a shoe, and *A*_n_ is the new, enlarged area, and the ratio of *A*_n_ to *A*_s_ minus 1 and times 100 corresponds to the percentage increase in heel width. When matching *PF*1 to *F*_max_, *F*_opt_ is replaced by *F*_max_ in Equations (2) and (3).

**Figure 26:**
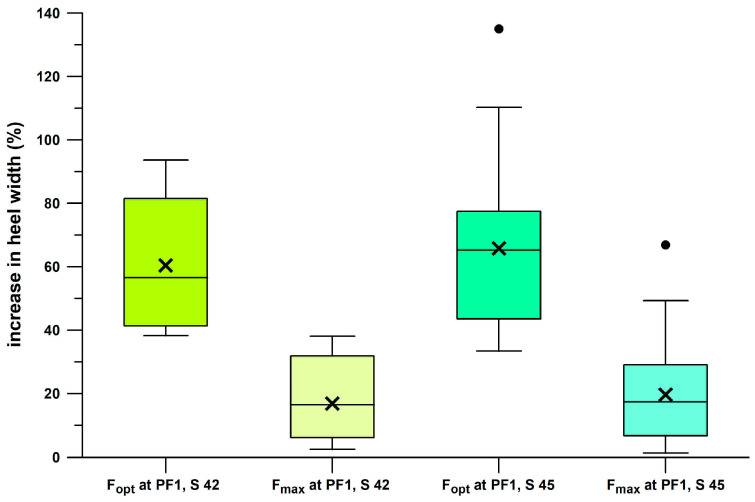
Boxplots of the percent increase in the heel width after matching F_opt_ (optimal force at the shoulder point) to PF1 (first peak of the vertical ground reaction force), and F_max_ (upper limit of forgiveness range) to PF1; S = shoe size (EU); ✖ = average; ● = outlier.

As shown in Figure 26, when *F*_max_ is set to *PF*1 for each participant and each shoe condition (when *PF*1 > *F*_max_ at the original heel size), the heel width needs to be increased by 16.83% (±12.30%) and 19.65% (±17.25%) for shoe sizes of 42 and 45, respectively. If *F*_opt_ is matched to *PF*1, the heel width needs to be increased by even 60.47% (±19.67%) and 65.79% (±27.53%), for shoe sizes of 42 and 45, respectively.

If the scaling factor is the same in all three dimensions, then a 10% increase in foot length means a 10% increase in forefoot width. However, the scaling factors are disproportionate, and, for example, the scaling factor of the forefoot width of the human foot is 0.67 on average (calculated from the data of Domjanic et al. [33]) relative to a foot length scaling factor of 1. Table 8 shows the relative scaling factors of the measured shoe parameters (six shoes of Cohort 3) relative to the shoe length (scaling factor of 1).

**Figure 27:**
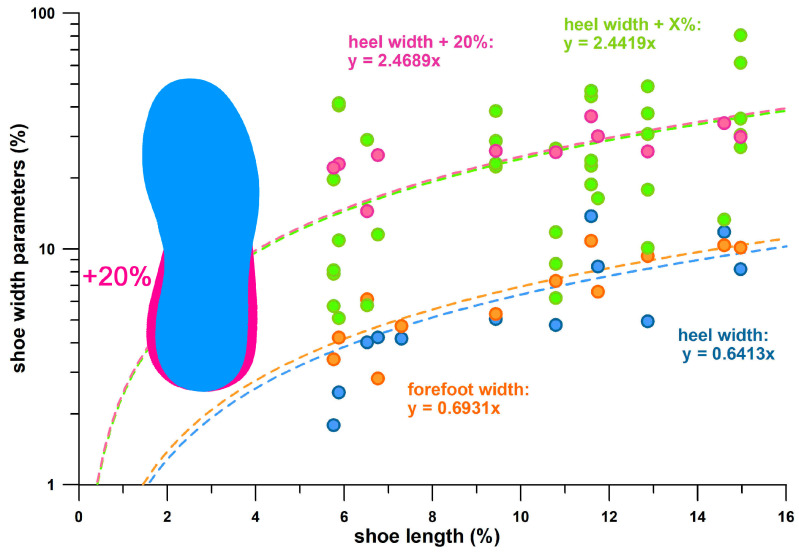
Increase in shoe width parameters (%) relative to shoe length versus increase in shoe length (%); x = shoe length (%); y = shoe width parameter (%); “y = 0.6413x” = the heel width increases by 6.4% if the shoe length increases by 10%; “+X%” = increase in heel width when matching F_max_ to *PF*1; dashed lines: linear fit functions corresponding to the four linear equations; inset: shoe print (blue) with heel enlarged by 20% (red).

The stack height had the smallest relative scaling factor of only 0.21. This seems to be a surprising result but is quite logical, considering that the stack height does not affect *F*_opt_ (Table 7). To adapt the heel design for heavier and taller users, the heel width must be changed as already explained above. The relative scaling factors of the forefoot and heel widths were 0.69 and 0.64, respectively (Table 8, and Figure 27). The forefoot shoe width scaling factor of 0.69 is close to that of the human forefoot width of 0.67. After increasing the measured heel widths by 20% (predicted maximum average value in Figure 27 for placement of *PF*1 at *F*_max_), the relative scaling factor of the heel width increases disproportionately to 2.44 (Table 8, and Figure 27). If the measured heel widths are increased according to Equation (3) such that *PF*1 = *F*_max_ for each individual whose *PF*1 was greater than *F*_max_, the relative scaling factor of the heel width increases disproportionately to 2.47 (Table 8, and Figure 26), i.e., very close to 2.44. This result confirms that increasing the heel width by 20% is a viable design strategy to reduce the number of outlier cases (Table 4 and Figure 26), i.e., large *PF*1 data outside the forgiveness ratio. Figure 27 also shows a standard shoe sole (blue) with a 20% increase in heel width (10% on either side).

## 4. Discussion

This study offers a new concept and method for evaluating the cushioning potential of athletic shoes. This method is independent of preset energy (ASTM [8,9]) or force values [21], and instead measures and examines the shoe-specific data at the optimum (“shoulder”) point of impact absorption, i.e., the maximum ratio of absorbed energy to applied force (*E*/*F*_max_). Thereby, ratios of high energy to low force are preferred. In addition, these ratios are related to the actual loading forces encountered at the heel during running. Excellent *E*/*F*_max_ values are useless if the corresponding force (*F*_opt_, at *E*/*F*_max_) is too low or too high. If the *F*_opt_ value is too low, the heel segment is under-designed; if it is too high, the heel is over-designed. This relationship connects the *E*/*F*_max_ value to the underlying design of the heel segment, which is likely an inadvertent or unwitting design, if the shoulder point principle is not known. In any case, the heel feels harder when running before or after the shoulder point (or at least outside of the forgiveness range), than when running at the shoulder point. The logical question to ask in this context is: do runners prefer to run at the shoulder point? This is the same question that came up in the introductory section of this article: do runners run with exactly 5 J of impact energy as required by ASTM test standards [8,9]? The counterargument to this is that poorly designed shoes, i.e., those not designed for running at the shoulder point, would prevent runners from running at that exact point. The present study provides at least partial evidence that runners run at the shoulder point (Table 4): at a running speed of 10 kph, the peak force at the heel (*PF*1) of all runners was within the forgiveness range in all shoes except the Joe Nimble shoe, whereas at individual running speed, only participants wearing Vivobarefoot and Puma Velocity shoes had their *PF*1 within the forgiveness range. The reason why many participants wearing any of the other four shoes at individual speed ran past the shoulder point was because they were too fast, in combination with being too heavy and too tall (Table 5 and Table 6), and, consequently, the shoes were not designed for these conditions.

Based on these results, our recommendation is to replace the ASTM test standards [8,9] with the new method. The new method uses floating parameters instead of preset values (such as the 5 J impact energy [8,9]). As already mentioned in the introductory section, other standards use similar methods to assess the hardness of foams, namely, the ISO and ASTM-C standards, which measure the stress of foams precisely at 40% and 50% strain, respectively. These preset strain values are unrealistic, as the optimum strain of shock absorbers (including foams) found by Fuss [14] ranged between 48.5% and 81%.

The new method presented in this article does not require realistic input parameters, and the heels of sports shoes can be compressed up to a force that allows us to find the shoulder point as well as the upper limit of the forgiveness range (*F*_max_). For most shoes, a force of 3 kN is sufficient. In addition, this method is not affected by the strain rate, so a deflection rate of 10 mm/s for 5 cycles suffices to obtain reliable results. A dynamic method, e.g., “*an 8.5-kg mass dropped from 50 mm*” [8,9], is therefore no longer required.

The energy absorption in the heel segment of a sports shoe under the new method does not imply a reduction in shock spike, or, generally, a reduction in ground reaction force. As already mentioned in the introductory section, the first force peak, *PF*1, of the vertical ground reaction force is not a transient spike, but rather represents the weightbearing force of the heel (Figure 1). When comparing two size 39 shoes, namely Vivobarefoot and Puma Liberate, the medians of *PF*1 did not differ significantly when running at 10 kph or at individual speed (10 kph: 0.96 kN and 0.92 kN, respectively; individual speed: 1.03 kN and 1.03 kN, respectively). These *PF*1 data were within the forgiveness range. However, the average energy absorbed by the heel segment of these two shoes at the shoulder point was completely different, namely, 0.6 J and 8 J for Vivobarefoot and Puma Liberate, respectively. What happens to the 7.4 J difference in energy absorption between Vivobarefoot and Puma Liberate that is not absorbed by Vivobarefoot? If the 7.4 J are not absorbed by the heel segment, this energy difference must be absorbed by the lower limbs. This means that the energy is dissipated in the body’s own tissues, such as muscles (e.g., through concentric contraction), tendons, bones, and joints (articular cartilage, ligaments). This redistribution of energy from the shoe sole to the human leg can lead to increased fatigue and more frequent overuse/overstrain syndromes. Therefore, the shoulder point parameters are ideal for shoe testing and selection. If a runner’s variation in *PF*1 is known, e.g., from previous force plate tests with different shoes, then diagrams such as Figure 10 and Figure 12 facilitate the informed selection of shoes with large *E*/*F*_max_ and large *E*_opt_ within the given *PF*1 window. In this context, the shoulder point parameters could initiate a healthy competition between the leading shoe companies to improve the cushioning properties of heel segments and promote the spirit of innovation.

**Figure 28:**
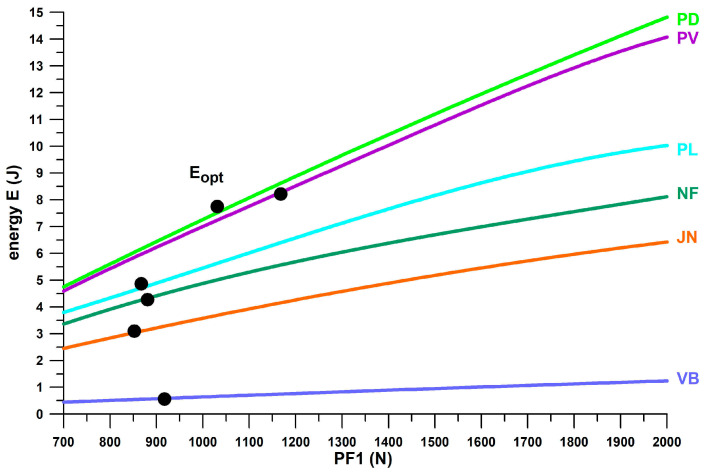
Energy absorbed vs. PF1 (first peak of the vertical ground reaction force); VB: Vivobarefoot PrimusLite, JN: Joe Nimble Addict, NF: Nike Free5.0, PL: Puma Liberate, PV: Puma Velocity2, PD: Puma DeviateElite 2; the black dots identify the optimum energy E_opt_ at maximum E/F (at the shoulder point).

Figure 28 addresses the problem with standards [8,9,10] and other methods [21], as mentioned in the Introduction section, and provides evidence regarding their applicability. Testing shoe heels at a fixed energy level of 5 J [8,9] is not applicable, as Puma Velocity and Deviate shoes absorb less than 5 J of energy only at a *PF*1 of less than 0.75 kN, and the energy absorbed by Vivobarefoot is less than 1.5 J up to 2 kN. Energy absorption therefore depends on the shoe type (the more minimalist and thinner the sole, the less energy is absorbed) and the *PF*1. A fixed force level of 5 kN [10] is not applicable, either, as the maximum *PF*1 determined in this study was less than 2 kN. Although a fixed force level of 1.5 kN [21] is well within the *PF*1 range determined in this study, *E*_opt_ lies in a window between 0.85 and 1.17 kN (Figure 28), and thus well before 1.5 kN.

Figure 9 and Figure 10 show a main line (blue dots) and a secondary line (red dots), with the shoes on the secondary line having an *E*/*F*_max_ ratio 1.6–1.4 higher than the shoes on the main line. Most of the shoes on the secondary line are PUMA shoes (Puma Deviate Elite 2, Puma Liberate, Puma Velocity 2, and PumaFastR), which have one thing in common: the midsole of these shoes is made from polymers saturated with supercritical CO_2_ under high pressure and temperature, which expands rapidly upon cooling or depressurization, resulting in a foam with a uniform cell structure, low weight, good cushioning, and high elasticity [34]. It is therefore likely that autoclave foaming results in better *E*/*F*_max_ ratios.

The forgiveness parameter *ϝ* describes the range between two force limits, *F*_min_ and *F*_max_, where *E*/*F* is greater than 97.5% of *E*/*F*_max_. This measure provides an optimum range rather than an optimum point. The larger the *ϝ*, the greater the force range in which the shoe shows its individual optimal and constant cushioning properties—or, conversely, the more compliant a heel sole segment is when exposed to widely varying *PF*1. However, despite its advantages, the forgiveness *ϝ* is not intended as a selection criterion of running shoes. For example, a rearfoot striker has a *PF*1 of 1.5 kN, due to his/her body mass, running speed, and running style. This value is within the optimum range for the Vivobarefoot heel (0.65–2.45 kN) but outside the optimum range for the Puma Liberate heel (0.70–1.25 kN). This does not mean that the athlete should choose the Vivobarefoot shoe over the Puma Liberate because it has a larger forgiveness range. Rather, the Puma Liberate should be preferred over the Vivobarefoot because it has a higher absolute energy absorption at *PF*1 (8.5 vs. 0.9 J). This comparison shows that forgiveness should be considered a supporting parameter for *E*/*F*_max_ and *F*_opt_. While *E*/*F*_max_ describes the performance of the shoe as an energy absorber, *F*_opt_ defines the design classification (Figure 10), and the forgiveness *ϝ* indicates the optimal force range within a given design classification. Nevertheless, there is evidence that the more minimalist a shoe is, the wider the forgiveness range is (Figure 23). Considering that heels of minimalist shoes are the worst energy absorbers due to their low stack height, and considering that shoes with less energy absorption result in lower *PF*1 anyway, as shown in Figure 24, a wider forgiveness range may better compensate for a randomly higher *PF*1.

The strategies for adjusting *F*_opt_ by adapting the shoe sole geometry in Section 3.9 are based on the principle that energy absorption increases linearly with the increase in heel volume. Thus, doubling the heel area increases energy absorption by a factor of two. This principle implies that the heel area of the human foot must increase by the same factor. However, if the human heel area remains constant but body mass increases, we can expect a non-linear, asymptotic rise in *F*_opt_ and *E*_opt_ as the sole area increases, based on the testing of foam sheets with different area sizes (Figure 20). This result is due to the fact that shoe soles are point-elastic and not area-elastic. We can therefore assume that adjusting the width of shoe heel will result in a limited adaptation of *F*_opt_.

The inclusion of shoes with 3D-printed midsoles was essential for the understanding of heel segment mechanics as well as for their practical application. Versions 1.1, with strut diameters of 16 mm, were the only under-designed shoes in this study (Figure 10). All 3D-printed midsoles (together with the Puma RuleBreaker shoe) exhibited the best *E*/*F*_max_ values of all shoes compressed at the same *x*_opt_ level (Figure 13). The additive manufacturing method enables the customised and personalised production of running shoes by simply adjusting the strut diameter of the unit cells. This principle was already illustrated in Figure 10 by shifting the under-designed shoes into the well-designed zone. Furthermore, the shoes identified in Table 4 and Figure 25, which were over-designed (beyond the shoulder point) at higher running speeds, can be easily adapted by increasing the strut diameter instead of increasing the heel area suggested for conventional running shoes (Figure 27). Increasing the stiffness of foam soles would be another option, but it is not suitable for the mass market. Increasing the strut diameter in 3D-printed midsoles stiffens the structure. Furthermore, the high-speed sintering (HSS) process with VX 200 (Voxeljet, Friedberg, Germany) allows for continuous variation of the stiffness within a 3D-printed object by controlling the selective energy input for particle fusion with infrared-absorbing ink in different shades of grey [35]. This specific process enables the integration of zones of different stiffness into the midsole of a running shoe, in addition to selectively changing the strut diameters. By selecting the optimal unit cell geometry and properties from hypothetical shape and material databases, a 3D-printed midsole could literally be customised (designed and manufactured) on-site to a runner, with the desired shoulder point parameters. There is limited literature on 3D-printed shoe midsoles which focuses on simulating plantar pressure distribution [36,37]. However, these studies did not address the cushioning properties and energy absorption of 3D-printed shoe midsoles.

A limitation of this new method is the repeatability of the shoulder point data. According to the repeatability tests, the uncertainty of *x*_opt_, *F*_opt_, *E*_opt_, and *E*/*F*_max_ was 3.24%, 6.01%, 6.32%, and 1.89%, respectively. The worst repeatability was seen in *F*_opt_ and *E*_opt_; however, although *E*/*F*_max_ is the ratio of *E*_opt_ to *F*_opt_, *E*/*F*_max_ had the least uncertainty. *E*/*F*_max_ is the most important shoulder point parameter. Testing a shoe multiple times (at least five times), with a 24 h rest between repetitions, and calculating the average values of the shoulder point parameters mitigates the problem.

## 5. Conclusions

This research study shows that existing standards [8,9], which simulate 5 J of energy absorption at the heel sole, are unsuitable because energy absorption depends on the construction of the heel sole and therefore cannot be a constant. The shoulder point method introduced in this study for running shoes identifies the optimal running point with maximum energy absorption at minimal force. Participants actually ran at the shoulder point (or at least within the forgiveness range) unless they were too heavy and ran at their preferred speed. The shoulder point parameters *E*/*F*_max_, *E*_opt_, *F*_opt_, and *x*_opt_ allow the classification of heel soles in terms of running before the shoulder point (over-designed), at the shoulder point (well-designed), and after the shoulder point (under-designed), thus assisting consumers in making informed choices. The shoulder point method also proved to be insensitive to strain rates, which improves shoe testing, as a standard compression testing machine is sufficient.

This research study proposes a revision of the current standards, and the adoption of the method described and explained above.

## Figures and Tables

**Table 1:** Overview of shoes investigated; HSS = high-speed sintering; LS = laser sintering; MJF = multi-jet fusion.

Nike	Puma	Adidas	Asics	Miscellaneous
Airmax	Accelerate Nitro SQD handball	Adizero	Gel	Bugatti
Airmax 90surplus	Clyde All-Pro Team basketball	Adizero Boston10	Gel Cumulus11	CEP Omnispeed Bowtech Bounce Prototype v3
Airmax BW	Defendor Running	Adizero F50	Gel DSTrainer	CEP Omnispeed Bowtech Bounce Prototype v5
Airmax Lunarlon	Deviate Elite 2	AdiZero Feather	Gel GT2150 Duomax	Guy Leech running shoe
Airmax270	Eliminate Nitro SQD handball	Boost	Gel Innovate5	Hylo Run3
Airmax270 w/o bladder	experimental (thick sole)	BounceTitan	Gel Kayano17	INov-8-roadx233
AirVisi Pro5	FastR	Fluidstreet	Gel Kayano18	Joe Nimble Addict
Flex Experience	Future	MegaBounce	Gel Kayano21	Karrimor Duma
Flex Experience RN3	Liberate	Nitrocharge	Gel Netburner	La Sportiva
FlyKnit One	Nrgy Star	Ozone3	Gel Nimbus14	Merrell Hiking Boot Refuge Pro GTX
FlyKnit Racer	RuleBreaker	SL20	Gel NoosaTri	NewBalance 1150
Free	TazonII	SpringBlade	GT1000	NewBalance 550
Free 5.0	Ultrawave prototype basketball	Supernova	Nimbus	On Cloudneo
Free Run	Vantage Nitro handball	TechSuper		Rieker
Free Run2	Velocity 2	Terrex 299		Saucony breakthru
Free Trail		TorsionSystem		Saucony Endorphin
Hypervenom		Yeezy Slide		Sketches Shape-Ups
Jordan1 Zoom	**Mizuno**	**3D-printed**	**Brooks**	Under Armour cartilage
Kobe Basketball	Rider	HSS soft	Adrenalin	Vibram Bikla
Lunar Edge	WaveCreation12	LS soft	Ghost11	Victor Badminton
React	WaveElixir4	MJF soft	Ghost13	Vivobarefoot Primus Lite
Renew	WaveInspire9	HSS hard	Ghost14	
Total90	WaveMusha3	LS hard	Ghost15	
Vomero7	WaveRider12	MJF hard	Glycerin11	
Zoom Hyperfuse			GTS	
ZoomAir			GTS11	

**Table 2:** Repeatability of six shoes expressed as the interquartile range (IQR) as a percentage; the data under “ALL” shoes correspond to those shown in Figure 19; the IQR data are colour coded in each row (red: high, green: low).

Shoulder Point Parameters	Adidas Mega-Bounce	Adidas SpringBlade	Brooks GTS11	ASICS Gel-Kayano17	Mizuno Wave Creation12	Nike Free	All
*E*/*F*_max_	2.05	2.24	5.83	3.78	2.12	1.35	2.88
*E*_opt_	14.21	5.22	5.99	8.91	15.34	4.22	8.59
*F*_opt_	14.79	2.68	3.69	5.05	8.67	2.83	11.64
*x*_opt_	2.13	9.06	5.01	3.43	1.65	4.59	5.25

**Table 3:** Shoulder point parameters and area of foam sheets.

Area (cm^2^)	*x*_opt_ (mm)	*F*_opt_ (kN)	*E*_opt_ (J)	*E*/*F*_max_ (J/kN)
25	13.53	0.693	3.50	5.05
36	14.01	0.838	4.28	5.11
49	14.70	0.905	4.63	5.11
64	14.88	0.957	4.82	5.04
81	14.97	1.011	5.05	4.99
100	14.94	1.003	4.95	4.94

**Table 4:** Number of participants who had the magnitude of their PF1 inside, or after the shoulder point; VB: Vivobarefoot PrimusLite, JN: Joe Nimble Addict, NF: Nike Free5.0, PL: Puma Liberate, PV: Puma Velocity2, PD: Puma DeviateElite 2.

Shoe	Before	Inside	After	Before	Inside	After
	Forgiveness Range (Speed 10 kph)			Forgiveness Range (Individual Speed)		
VB	0	10	0	0	16	1
JN	0	6	**4**	0	8	**9**
NF	0	9	1	1	7	**9**
PL	0	8	2	0	7	**10**
PV	0	10	0	1	14	2
PD	0	9	1	1	8	**8**

**Table 5:** Comparison of participant-specific attributes for 2 groups: PF1 before or inside the forgiveness range (*n* = 64), and PF1 past the forgiveness range (*n* = 38); data for individual speed only, as 10 kph running speed showed a negligible number of outliers; PF1 = first force peak of the vertical ground rection force; F_max_ = upper boundary of the forgiveness range; *p* = *p*-value; U = U-statistic of the Mann–Whitney test; r = effect size.

	Speed (kph)	Body Weight (N)	Body Height (m)	BMI (kg/m^2^)	PF1 (N)	Shoe Size	Gender (0 = f, 1 = m)
median (PF1 **<** F_max_)	12.0	593.5	1.740	20.84	1080.2	2.00	0
median (PF1 **>** F_max_)	12.4	760.3	1.785	24.664	1504.7	2.50	1
average (PF1 **<** F_max_)	11.6	631.5	1.741	21.10	1077.4	1.77	0.4375
average (PF1 **>** F_max_)	12.3	767.8	1.785	24.52	1517.8	2.55	1
*p*	0.0023	<0.0001	0.0074	<0.0001	<0.0001	<0.0001	<0.0001
U_min_	775	430	828	320	189	574	532
r	0.3627	0.6464	0.3191	0.7368	0.8446	0.5280	0.5625
effect size	medium	large	medium	large	large	large	large

**Table 6:** Averages of participant’s attributes for three shoe sizes and three running speeds.

Size	Velocity (kph)	Body Weight (N)	Body Height (m)	PF1 (N)
all velocities				
small (EU 39)	10.44	584.9	1.680	984.2
medium (EU 42)	11.18	691.7	1.761	1179.1
large (EU 45)	11.50	767.5	1.798	1417.8
10 kph running velocity				
small (EU 39)	10	607.6	1.696	946.3
medium (EU 42)	10	715.5	1.765	1153.5
large (EU 45)	10	760	1.793	1341.6
individual running speed				
small (EU 39)	9.18	609.7	1.681	1034.7
medium (EU 42)	12.34	687.6	1.763	1257.2
large (EU 45)	12.49	770.3	1.799	1510.4

**Table 7:** Shoulder point data of Puma Deviate Elite; standard: original data; stack + 15%: change of the shoulder point data when increasing the stack height by 15%; area + 15%^2^ each: change in the shoulder point data when increasing the heel length and width by 15%; x_opt_, F_opt_, E_opt_ = deflection, force, and energy at the shoulder point; E/F_max_ = maximum ratio of energy to force.

Size	Standard	Stack + 15%	Area + 15%^2^
*x*_opt_ (mm)	23.4	**26.9**	23.4
*F*_opt_ (kN)	1.06	1.06	**1.40**
*E*_opt_ (J)	8.10	**9.32**	**10.69**
*E*/*F*_max_ (J/kN)	7.64	**8.79**	7.64

**Table 8:** R^2^ of linear regression (increase in shoe parameters relative to shoe length versus increase in shoe length) explains by how much (percentage if R^2^·100) the increase in a dependent shoe parameter (relative to the shoe length) is explained by the increase in the independent shoe length (linear regression); *p* of R^2^ indicates whether the apparent trend of the regression slope is real, i.e., positive slope significantly different from a zero slope or a negative slope, if *p* ≤ 0.1 (one-tailed, alpha = 0.1); the scaling factor is the regression slope trough origin, relative to a shoe length scaling factor of 1.

Shoe Parameter Relative to Shoe Length	R^2^ of Linear Regression	*p* of R^2^	Relative Scaling Factor (Slope Through Origin)
stack height	0.1918	0.0690	0.2130
forefoot width	0.9316	<0.0001	0.6931
heel width 1 (measured)	0.7644	<0.0001	0.6413
heel width 2 (increase in measured width by 20%)	0.2324	0.0022	2.4419
heel width 3 (increase in measured width such that PF1 = F_max_)	0.4629	<0.0001	2.4689

## Data Availability

The data presented in this study are available on request from the first author to any qualified researcher who has obtained Ethics Approval for secondary use of existing data through a Consent Waiver.
